# Supplementary material for: A Transient Translaminar GABAergic Interneuron Circuit Connects Thalamocortical Recipient Layers in Neonatal Somatosensory Cortex
Source: Neuron. 2016 Feb 3;89(3):536–49. doi: 10.1016/j.neuron.2016.01.015 (PMC4742537; doi:10.1016/j.neuron.2016.01.015)
Supplement: Document S1. Figures S1–S4 and Tables S1 and S2 [file mmc1.pdf]

Neuron

Supplemental Information

A Transient Translaminar GABAergic Interneuron Circuit Connects Thalamocortical Recipient Layers in Neonatal Somatosensory Cortex

Andre Marques-Smith, Daniel Lyngholm, Anna-Kristin Kaufman, Jacqueline A. Stacey, Anna Hoerder-Suabedissen, Esther B.E. Becker, Michael C. Wilson, Zoltán Molnár, and Simon J.B. Butt

SUPPLEMENTAL FIGURES

Figure S1 [related to Figure 3 and Figure 4]. Laminar organisation of normalised glutamatergic input onto Lpar1-INs and GABAergic input onto L4 SSNs

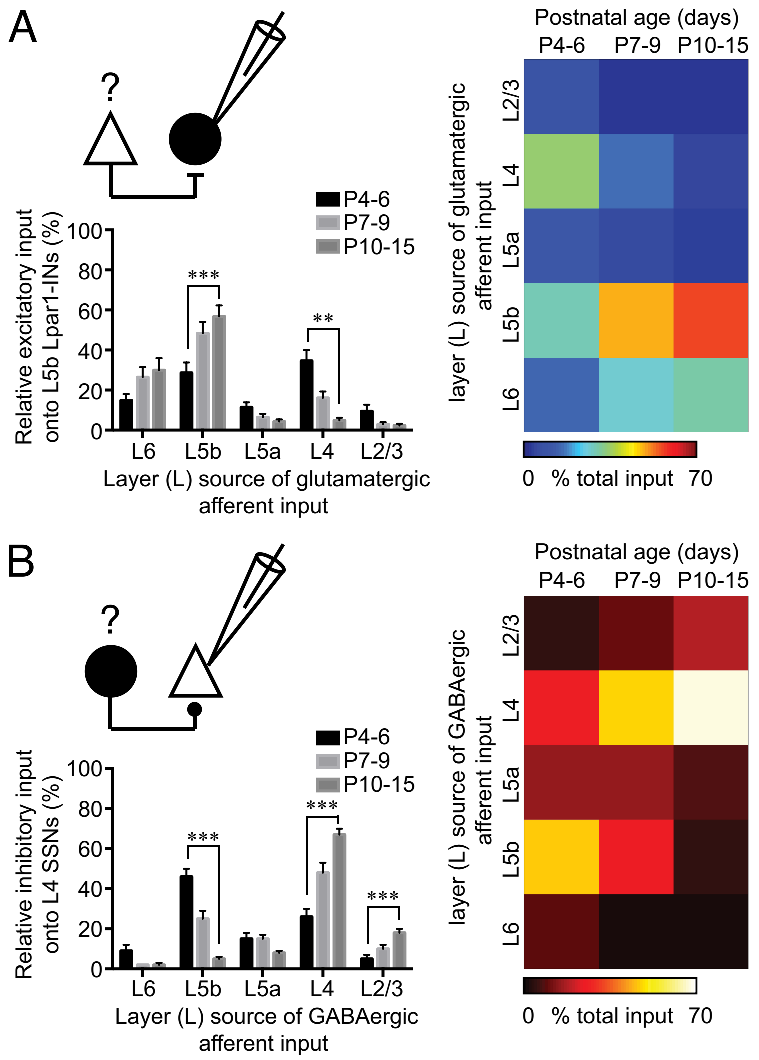

(A) *Left*, percentage afferent excitatory input from each layer onto Lpar1-INs (schematic black circle) as revealed by LSPS of caged glutamate. Local L5b synaptic input increased significantly between P4-6 and P10-15 (Kruskal-Wallis test, H(3, 29) = 9.86, $p = 0.007$; Dunn's multiple comparisons test (Dmc), $p = 0.002$). L4 input decreased significantly (Kruskal-Wallis test, H(3, 29) = 13.50, $p = 0.001$; Dmc, $p = 0.0002$). Error bars are ±SEM. *Right*, summary plot showing the developmental remodelling of afferent excitatory laminar input sources onto Lpar1-INs through development.

(B) *Left*, percentage afferent GABAergic input per layer revealed by LSPS of caged glutamate and holding the postsynaptic L4 SSN at E_{Glut}. Input originating from L5b decreased significantly between P4-6 and P10-15 (Kruskal-Wallis test, H(3, 51) = 30.9, $p = 0.0001$; Dunn, $p = 0.001$), as did L5a input (Kruskal-Wallis test, H(3, 51) = 8.43, $p = 0.015$; Dunn, $p = 0.054$). L4 input significantly increased over the same period (Kruskal-Wallis test, F(3, 51) = 31.4, $p = 0.0001$; Dunn, $p = 0.0001$), as did L2/3 input (Kruskal-Wallis test, H(3, 51) = 20.3, $p = 0.0001$; Dunn, $p = 0.001$). Error bars are +/-SEM. *Right*, summary plot of the developmental remodelling of afferent GABAergic input onto Lpar1-EGFP INs as arranged by laminar sources.

Figure S2 [related to Figure 5]. Infraorbital nerve (ION) transection reduces total glutamatergic input onto L5b Lpar-INs.

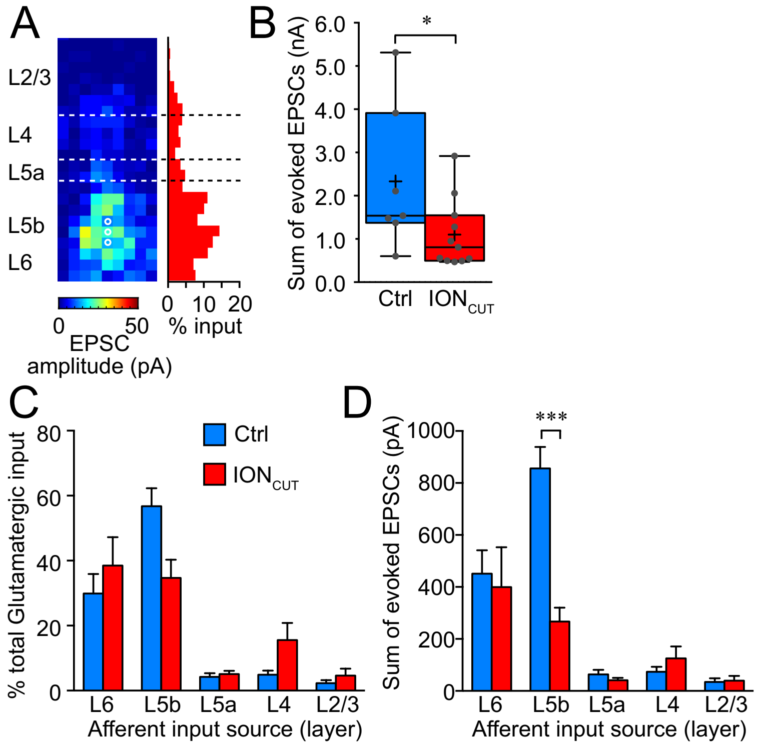

(A) Average map (left) of evoked glutamatergic input onto Lpar1-INs (n=11) in ION transected (ION_{cut}) animals at P10-15. *Right panel*, normalised profile of glutamatergic input onto ION_{cut} Lpar1-INs across the depth of the cortical column.
(B) Total glutamatergic input onto Lpar1-INs at P10-15 is significantly reduced (*p=0.020, Mann-Whitney test, U(82,108)=16) in IONcut animals compared to controls.
(C) Normalised glutamatergic input onto Lpar1-INs showed no layer difference in distribution between IONcut and control at P10-15.
(D) Total glutamatergic input on Lpar1-INs in IONcut animals broken down according to layer. The decrease in total input from L5b (***p<0.0001, Mann-Whitney test, U(69,141)=3.00) was not accompanied by a compensatory increase in other cortical layers.

Figure S3 [related to Figure 6]. Experimental strategy employed to examine the impact of conditionally silencing SST+ cells on the transient L5b-L4 GABAergic connection.

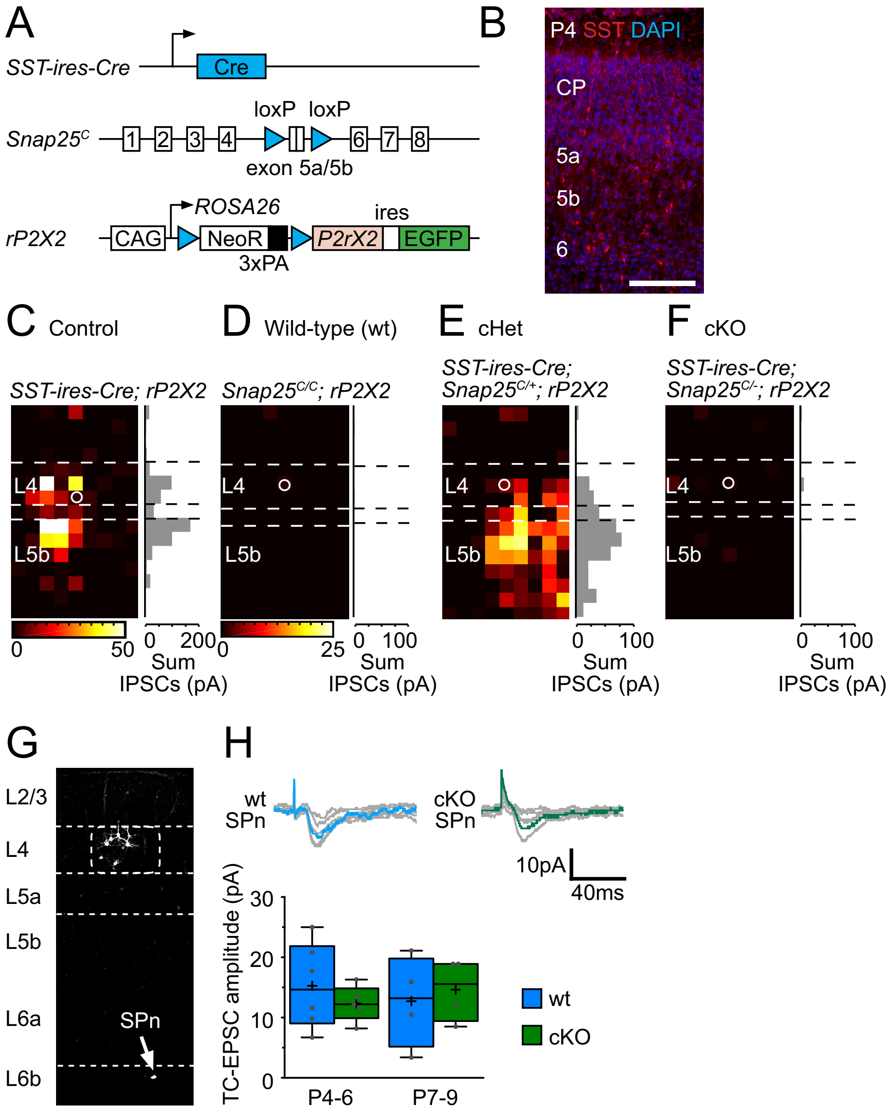

(A) The genetic strategy for examining Snap25 conditional loss-of-function while simultaneously enabling LSPS stimulation of SST+ cells. Males with the SST-ires-Cre driver and a single copy of the floxed Snap25 (*Snap25^{C}*) allele were crossed to females that were homozygote for the floxed Snap25 allele and a floxed-stop rat P2X2 receptor (rP2X2) with bicistronic ires-EGFP for genetic fate mapping. Unfortunately EGFP expression was not detected in SST+ cells either by visual inspection in acute *in vitro* slices or following immunohistochemistry.
(B) The presence of SST+ cells in P4 somatosensory cortex in conditional loss-of-function Snap25 (cKO) animals.
(C-F) Use of the P2X2 optogenetic strategy enables us to determine the source of SST+ cell input onto SSNs in the developing neocortex and confirm an absence of SST+ IN signalling in Snap25 cKO pups.
(C) LSPS map data from a P5 SSN in a control animal conditionally expressing rP2X2 in cells expressing Cre under the SST-ires-Cre driver line. UV laser uncaging of ATP evoked action potentials in cells expressing the rP2X2 receptor enabling LSPS mapping of input onto the SSN (left panel). Right panel, distribution of input onto the SSN arising from SST+ cells. Dashed white lines, layer boundaries; white circle, cell location.
(D) No GABAergic input was mapped onto L4 SSNs following ATP uncaging in animals that did not express Cre. The example shown is a recording from a L4 SSN (white circle) in an acute *in vitro* slice preparation of a P5 Snap25$^{C/C}$;rP2X2 pup.

(*Cont.*)

(E) LSPS map of ATP uncaging-evoked responses onto a L4 SSN in a P6 conditional Snap25 heterozygote (cHet) pup. Scale bar is the same as shown in panel (D).
(F) Failure to observe ATP uncaging-evoked GABAergic inputs onto a L4 SSN in a conditional Snap25 knockout (cKO) P5 animal.
(G) Recovered morphologies of L4 SSNs and a single nearby subplate neuron (SPn) in a P7 wild-type acute *in vitro* thalamocortical slice. Recordings of L4 SSNs were only performed in animals in which thalamocortical connectivity into the neocortex was confirmed by preservation of input onto SPns (see panel H).
(H) Electrical stimulation of the VPM evoked TC-EPSCs in subplate (SPn) neurons in both wild-type (wt; blue average trace) and Snap25 conditional knockout (cKO) animals (both P7). *Bottom graph*, amplitude of TC-EPSCs recorded under voltage clamp (hp: -70mV) in SPn.

Figure S4 [related to Figure 7]. Over-expression of Nrg-1type1 protein in early postnatal development does not result in a change of spontaneous synaptic activity recorded from L4 neurons.

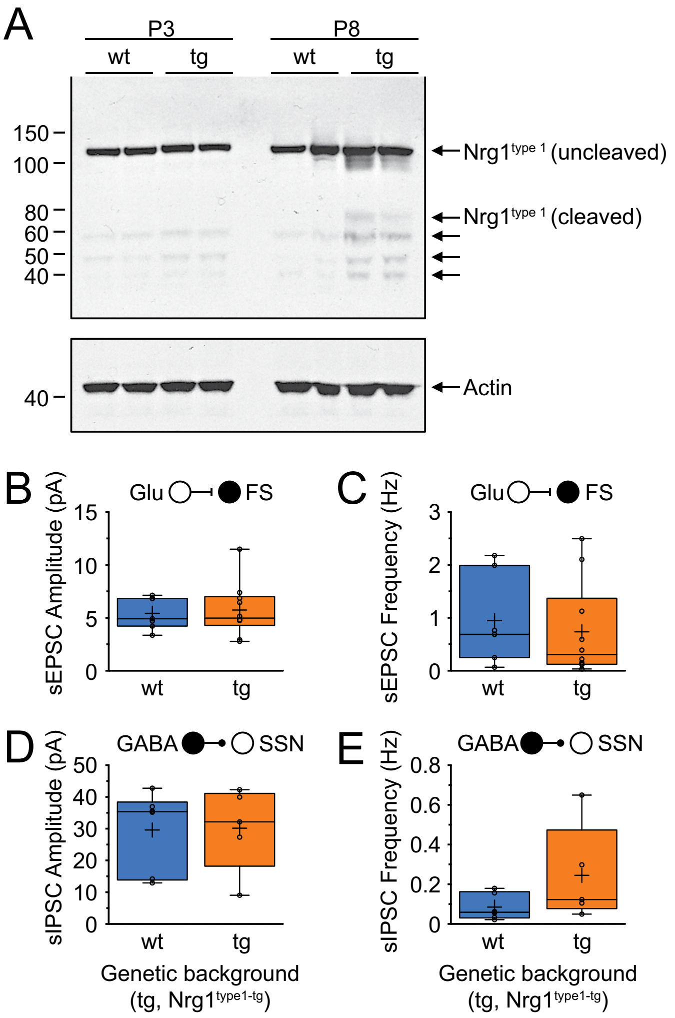

(A) Western blot for Ig-Neuregulin-1 (type 1) using cortical tissue from P3 and P8 animals in wild-type (wt) and over-expression Nrg-1type1-tg (tg) littermates. β Actin was used as loading control.
(B) Amplitude of spontaneous excitatory postsynaptic currents (sEPSCs)(hp:-70mV) recorded in layer 4 fast spiking (FS) interneurons arising from glutamatergic (Glu) afferent input; wt, n=7 cells from 6 animals (P4-P7); tg, n=10 cells from 9 animals (P4-P7).
(C) Frequency of sEPSCs (hp:-70mV) recorded in the same FS INs as panel (A)
(D,E) Corresponding data for spontaneous inhibitory postsynaptic currents recorded in L4 spiny stellate neurons (SSNs) arising from afferent input from GABAergic INs; wt, n=6 cells from 4 animals (P4-P7); tg, n=6 cells from 4 animals (P4-P7).

SUPPLEMENTAL TABLES

Table S1 [related to Figure 1]. Maturation of the intrinsic electrophysiological properties of S1BF Lpar1-INs.

	**P4-P6 (n = 12)**	**P7-P9 (n = 14)**	**P10-P15 (n = 29)**
**RMP (mV)**	-58.0 ±2.1	-56.3 ±1.0	-56.6 ±0.8
*****R_{in} (MΩ)**	1349 ±109	529 ±56	372 ±51
*****Rheobase (pA)**	8± 1.0	17 ±2	30± 4
*****Tau (ms)**	78.5 ±5.4	36.4 ±3.4	31.5 ±3.0
***AP threshold (mV)**	-39.5 ±1.0	-43.0 ±0.9	-42.7 ±3.1
****AP height (mV)**	58.5 ±2.8	68.7 ±2.9	69.6 ±1.7
*****AP HW (ms)**	3.0±0.2	1.8±0.1	1.3±0.1
***Adaptation (%)**	19± 5	22± 3	30± 2
***sAHP time (ms)**	37.1± 3.7	24.2± 2.7	25.9± 3.1
**sAHP amp. (mV)**	14.1± 0.5	13.7± 0.8	13.5± 1.1
**fAHP time (ms)**	-	-	5.9± 0.4
**fAHP amp. (mV)**	-	-	11.1± 1.2
**Voltage sag (mV)**	1.8± 1.6	5.5± 0.7	6.4± 0.50
*****Max. Freq. (Hz)**	38± 3	53± 3	70± 4

Table S1 abbreviations: RMP, resting membrane potential; R_{in}, input resistance; AP, action potential; AHP, after-hyperpolarisation. Values reported in each cell are average± SEM. Asterisks indicate one-way ANOVA or Kruskal-Wallis test *p* values: * $p< 0.05$; ** $p< 0.01$; *** $p< 0.001$.

Table S2 [related to Figure 5]. Intrinsic excitability of L4 fast-spiking and L5b Lpar1-INs in control and ION_{CUT} groups at P10-P15.

	**L4 FS INs P10-P15**		**L5b Lpar1-INs P10-P15**	
	**Control n = 15**	**ION_{CUT} n = 8**	**Control n = 29**	**ION_{CUT} n = 12**
**RMP (mV)**	-60 ± 1	-62 ± 2	-57 ±1	-59 ± 1
**R_{in} (MΩ)**	194 ± 16	281 ± 26*	372 ±51	455 ± 73
**Rheobase (pA)**	127 ± 20	81 ± 25	30± 4	21 ± 4
**AP threshold (mV)**	-31 ± 1	-32 ± 1	-43 ±3	-43 ± 1
**Max. Freq. (Hz)**	122 ± 8	101 ± 13	70± 4	79 ± 4
**Tau (ms)**	26 ± 4	17 ± 1	32 ±3	30 ± 2

Table S2 abbreviations as in Table S1. Values reported in each cell are mean ±SEM. Asterisks indicate Student's t test or Mann-Whitney test significance *p* values: ** $p< 0.01$. Statistically significant differences were only found for Input Resistance between Control and ION_{CUT} L4 FS INs (Student's t test, t (21) = 2.82, $p = 0.010$).
