# Supplementary material for: A Transient Translaminar GABAergic Interneuron Circuit Connects Thalamocortical Recipient Layers in Neonatal Somatosensory Cortex
Source: Neuron. 2016 Feb 3;89(3):536–49. doi: 10.1016/j.neuron.2016.01.015 (PMC4742537; doi:10.1016/j.neuron.2016.01.015)
Supplement: Document S2. Article plus Supplemental Information [file mmc2.pdf]

Article

Neuron

A Transient Translaminar GABAergic Interneuron Circuit Connects Thalamocortical Recipient Layers in Neonatal Somatosensory Cortex

Highlights

- Early postnatal thalamic synaptic input onto L5b somatostatin interneurons
- Transient reciprocal connectivity between L5b INs and L4 spiny stellate cells
- Sensory activity is required for the transition to a local L4 GABAergic circuit
- Molecular bias toward early local IN synapses delays thalamic innervation of SSNs

Authors

Andre Marques-Smith, Daniel Lyngholm, Anna-Kristin Kaufmann, ..., Michael C. Wilson, Zoltán Molnár, Simon J.B. Butt

Correspondence

simon.butt@dpag.ox.ac.uk

In Brief

Marques-Smith et al. identify a transient, early postnatal GABAergic interneuron circuit involved in thalamocortical integration in primary somatosensory cortex. Perturbation of sensory and molecular cues alters the temporal dynamics of this circuit and thereby maturation of thalamocortical afferent input.

Marques-Smith et al., 2016, Neuron *89*, 536–549
February 3, 2016 ©2016 The Authors
http://dx.doi.org/10.1016/j.neuron.2016.01.015

CrossMark

CellPress

Neuron
Article

A Transient Translaminar GABAergic Interneuron Circuit Connects Thalamocortical Recipient Layers in Neonatal Somatosensory Cortex

Andre Marques-Smith,[1,3,4] Daniel Lyngholm,[1,3,5] Anna-Kristin Kaufmann,[1,6] Jacqueline A. Stacey,[1] Anna Hoerder-Suabedissen,[1] Esther B.E. Becker,[1] Michael C. Wilson,[2] Zoltán Molnár,[1] and Simon J.B. Butt[1,*]
[1]Department of Physiology, Anatomy, and Genetics, University of Oxford, Oxford OX1 3QX, UK
[2]Department of Neurosciences, University of New Mexico Health Sciences Center, Albuquerque, NM 87131, USA
[3]Co-first author
[4]Present address: MRC Centre for Developmental Neurobiology, King's College London, Guy's Hospital Campus, London SE1 1UL, UK
[5]Present address: Strathclyde Institute of Pharmacy & Biomedical Science, University of Strathclyde, Glasgow G4 0RE, UK
[6]Present address: MRC Brain Network Dynamics Unit, Mansfield Road, Oxford OX1 3TH, UK
*Correspondence: simon.butt@dpag.ox.ac.uk
http://dx.doi.org/10.1016/j.neuron.2016.01.015
This is an open access article under the CC BY license (http://creativecommons.org/licenses/by/4.0/).

SUMMARY

GABAergic activity is thought to influence developing neocortical sensory circuits. Yet the late postnatal maturation of local layer (L)4 circuits suggests alternate sources of GABAergic control in nascent thalamocortical networks. We show that a population of L5b, somatostatin (SST)-positive interneuron receives early thalamic synaptic input and, using laser-scanning photostimulation, identify an early transient circuit between these cells and L4 spiny stellates (SSNs) that disappears by the end of the L4 critical period. Sensory perturbation disrupts the transition to a local GABAergic circuit, suggesting a link between translaminar and local control of SSNs. Conditional silencing of SST+ interneurons or conversely biasing the circuit toward local inhibition by overexpression of neuregulin-1 type 1 results in an absence of early L5b GABAergic input in mutants and delayed thalamic innervation of SSNs. These data identify a role for L5b SST+ interneurons in the control of SSNs in the early postnatal neocortex.

INTRODUCTION

The role of sensory experience and electrical activity on the development and refinement of neuronal circuits has long been one of the fundamental questions of neurobiology (Katz and Shatz, 1996). Seminal studies showed that early lesions of the sensory periphery have long-lasting consequences on the structural organization of cortical areas responsible for sensory processing (Hubel and Wiesel, 1964, 1970; Van der Loos and Woolsey, 1973). Thalamic nuclei provide the essential link between sensory periphery and the neocortex, with recent studies demonstrating that activity relayed to the developing neocortex by these nuclei has a crucial role in shaping lamination, neuronal morphology, and circuit organization (Chou et al., 2013; De la Rossa et al., 2013; Li et al., 2013; Matsui et al., 2013; Pouchelon et al., 2014; Vue et al., 2013). While early thalamocortical activity has been consistently observed in the form of spindle bursts (SBs) and early gamma oscillations (EGOs) as early as postnatal day (P)1 in vivo (An et al., 2014; Khazipov et al., 2004; Minlebaev et al., 2011; Yang et al., 2009, 2013a, 2013b), much less is known about the cortical circuits that are in place to receive and process thalamic input with the exception of transient circuits formed by subplate neurons (SPNs).

SPNs form a distinct layer between the white matter and cortical plate (CP), present early in development but largely eliminated by adulthood (Allendoerfer and Shatz, 1994; Kanold and Luhmann, 2010). SPNs receive robust early input from the thalamus, neuromodulatory systems, and excitatory and inhibitory neurons in the CP (Hanganu et al., 2002; Higashi et al., 2002; Zhao et al., 2009) and form a dense recurrent network mediated by chemical and electrical connectivity (Dupont et al., 2006). This local network has been proposed to function as an amplifier of thalamic and neuromodulatory input (Luhmann et al., 2009), coordinating activity, and regulating ocular-dominance plasticity (Kanold and Shatz, 2006), as well as early rhythmic activity (Dupont et al., 2006; Tolner et al., 2012) in the CP via projections that span its entire depth (Friauf et al., 1990; Zhao et al., 2009). Critically, these studies highlight the distinct nature of the developing brain and identify that other cell types—notably GABAergic interneurons (INs) (Luhmann et al., 2014)—also play a role in such early transient circuits.

In the neocortex, GABAergic synapses are first identifiable at embryonic day E16 (König et al., 1975), and spontaneous inhibitory postsynaptic currents (IPSCs) can be recorded in pyramidal cells (PYRs) as early as E18, with the majority of PYRs exhibiting IPSCs by P5 (Owens et al., 1999; Verhage et al., 2000). Consistent with these findings, paired-recording experiments have demonstrated that connectivity between fast-spiking (FS) INs and PYRs emerges around P5, but that the connection probability remains relatively low until P8–P10, at which point there is a further increase in connectivity rate (Daw et al., 2007; Pangratz-Fuehrer and Hestrin, 2011). This second step coincides with thalamic engagement of layer (L)4 FS cells in somatosensory whisker barrel cortex

(Daw et al., 2007) and is driven by sensory activity-dependent mechanisms (Chittajallu and Isaac, 2010). The delayed engagement of FS cells fits with the transition from the SPN-dominated early circuit to a requirement for tighter temporal control of activity in mature cortical circuits, yet it is also evident that GABAergic neurotransmission plays an important role within the first postnatal week (Ben-Ari et al., 2004), including in the developing thalamocortical network. Polysynaptic IPSCs can be evoked in granular and infragranular neocortical neurons following electrical stimulation of thalamic afferents from birth onward (Agmon et al., 1996). Moreover, in vivo blockade of GABAergic transmission increases the rate of occurrence, prolongs the duration, and expands the spatial spread of SBs and EGOs (Minlebaev et al., 2007, 2009, 2011). Taken together, these data suggest that GABAergic circuits are present and participate in early sensory-evoked activity prior to the emergence of canonical feedforward inhibition observed in L4, mediated by FS parvalbumin-positive (PV+) INs (Daw et al., 2007). However, the identity of these circuits is not known.

To better understand early thalamocortical networks, we have performed recordings in a transgenic mouse line, *Lpar1-EGFP*, which labels a population of SPNs (Hoerder-Suabedissen and Molnár, 2013) and infragranular somatostatin-positive (SST+) INs. We hypothesized that the latter—early-born INs located in deep cortical layers (Miyoshi et al., 2007)—might be the early GABAergic component of the thalamocortical network. We observed that L5b *Lpar1-EGFP* INs are targeted by direct thalamic input similar to SPNs (Friauf et al., 1990; Higashi et al., 2002) but also receive transient innervation from L4 excitatory neurons. At the same time, L4 SSNs are dominated by GABAergic input from L5b. We reveal that the transition from early translaminar L5b to mature local GABAergic innervation of SSNs requires normal sensory experience and can be perturbed by genetic silencing of SST+ INs and through genetic manipulation of a molecular signaling pathway thought to be important for the establishment of local PV-SSN synaptic connectivity (Fazzari et al., 2010). These data identify a transient L5b GABAergic input into L4 that is dismantled following the timely acquisition of thalamocortical synapses by SSNs.

RESULTS

Identification of Lpar1-EGFP Infragranular SST+ INs in the Developing Neocortex

The *Lpar1-EGFP* transgenic mouse labels SPNs in L6b (Hoerder-Suabedissen and Molnár, 2013) and a population of LIM homeobox transcription factor Lhx6-positive (P8, Lhx6+/EGFP+: 74% ± 5%; Figure 1A) infragranular (Figure 1B) cells with bitufted somatodendritic morphology. Immunohistochemistry revealed that these cells expressed SST at both early (P8, SST+/EGFP+: 72% ± 7%; Figure 1C) and late ages (P15, SST+/EGFP+: 82% ± 4%; Figure 1D), but not PV (P15, PV+/EGFP+: 1% ± 1%; Figure 1E), consistent with bitufted INs originating from the *Nkx2-1* domain of the ventral telencephalon (Butt et al., 2005; Miyoshi et al., 2007). Overall, EGFP cells accounted for 46% ± 8% of SST+ cells across the depth of the cortex at P15 (n = 4 animals) and 85% ± 7% of SST+ cells within L5b. *Lpar1-EGFP* neurons in L6b did not express IN markers (Figures 1A, 1D, and 1E), in line with previous data (Hoerder-Suabedissen and Molnár, 2013). To further determine the identity of the INs (Lpar1-INs), we performed whole-cell patch-clamp recordings in acute in vitro thalamocortical slice preparations. Intrinsic electrophysiological properties of early (< P7; Figures 1F and 1G) and juvenile L5b Lpar1-INs (P7+; Figures 1H and 1I) were characteristic of non-fast spiking (NFS) INs (see Table S1 available online) (Miyoshi et al., 2007; Wang et al., 2004). Recovered morphologies of L5b cells (n = 15) revealed that they possessed ascending axons which—when preserved in the slice—extended to L1 (Figures 1F and 1H). These data suggest that the *Lpar1-EGFP* transgene labels a population of infragranular, predominantly L5b, SST+, NFS IN throughout early postnatal development.

Lpar1-INs Receive Facilitating Thalamic Input

Similar to mature NFS INs (Cruikshank et al., 2010; Porter et al., 2001; Tan et al., 2008), L5b Lpar1-INs in the whisker barrel cortex (S1BF) received afferent thalamic input (Figure 2A) throughout the early period of development studied. Electrical stimulation of the VPM or internal capsule (IC) in acute in vitro somatosensory thalamocortical (TC) slices (Agmon and Connors, 1991) resulted in small-amplitude, short-latency (~10 ms) EPSCs (Figures 2A–2C) in the majority of recorded L5b Lpar1-INs (P4–P6, 11/18 cells; P7–P9, 11/13; P10–P15, 15/18). Using a minimal stimulation protocol (Gil and Amitai, 1996; Gil et al., 1999; Raastad et al., 1992), we found that EPSC amplitude remained constant over the first 2 postnatal weeks (Figure 2B), whereas latency of EPSC onset decreased (Figure 2C) (Salami et al., 2003). To further examine the origin of the electrically evoked EPSCs, we tested the effect of a 5-HT_{1B} agonist, CP93129, previously shown to selectively suppress early neonatal TC-EPSCs (Crocker-Buque et al., 2015). Ten minutes of perfusion with CP93129 (100 μM) suppressed evoked EPSC amplitude to 51% ± 6% of control (n = 6) (Figure 2D), consistent with the EPSCs having a thalamocortical (TC-EPSCs) as opposed to corticothalamic origin (Crocker-Buque et al., 2015).

To examine the short-term plasticity of this thalamic input onto L5b Lpar1-Ins, we repeatedly evoked TC-EPSCs using minimal electrical stimulation of the VPM across development (Figures 2E–2G). At later ages (P7+), repeat stimulation (10–40 Hz) resulted in a larger response to the second stimulation (Figure 2E), as reported for mature INs (Tan et al., 2008), and continued to augment in response to further stimuli, contrary to previously observed corticothalamic inputs onto infragranular SST+ INs (West et al., 2006). Under current clamp conditions, paired stimuli were sufficient to drive action potentials (APs) in Lpar1-INs (Figure 2F). Short-term plasticity (paired-pulse ratio, PPR) of TC input was not observed in P4-6 Lpar1-INs but over development became progressively more facilitating (Figure 2G). The emergence of short-term facilitating TC input onto Lpar1-IN was in contrast to other TC-recipient cell types (see also Tan et al., 2008), which remained constant in their response over the time period studied (Figure 2H). TC responses recorded in L6b *Lpar1-EGFP* SPNs showed short-term depression, whereas L4 SSNs and FS INs exhibited no short-term plasticity early in development. As such, Lpar1-INs were distinct in receiving short-term facilitating input during the L4 critical period plasticity (CPP) (Figures 2G and 2H). These data identify Lpar1 SST INs as a target for early TC innervation, with connections maturing over the first 2 postnatal weeks.

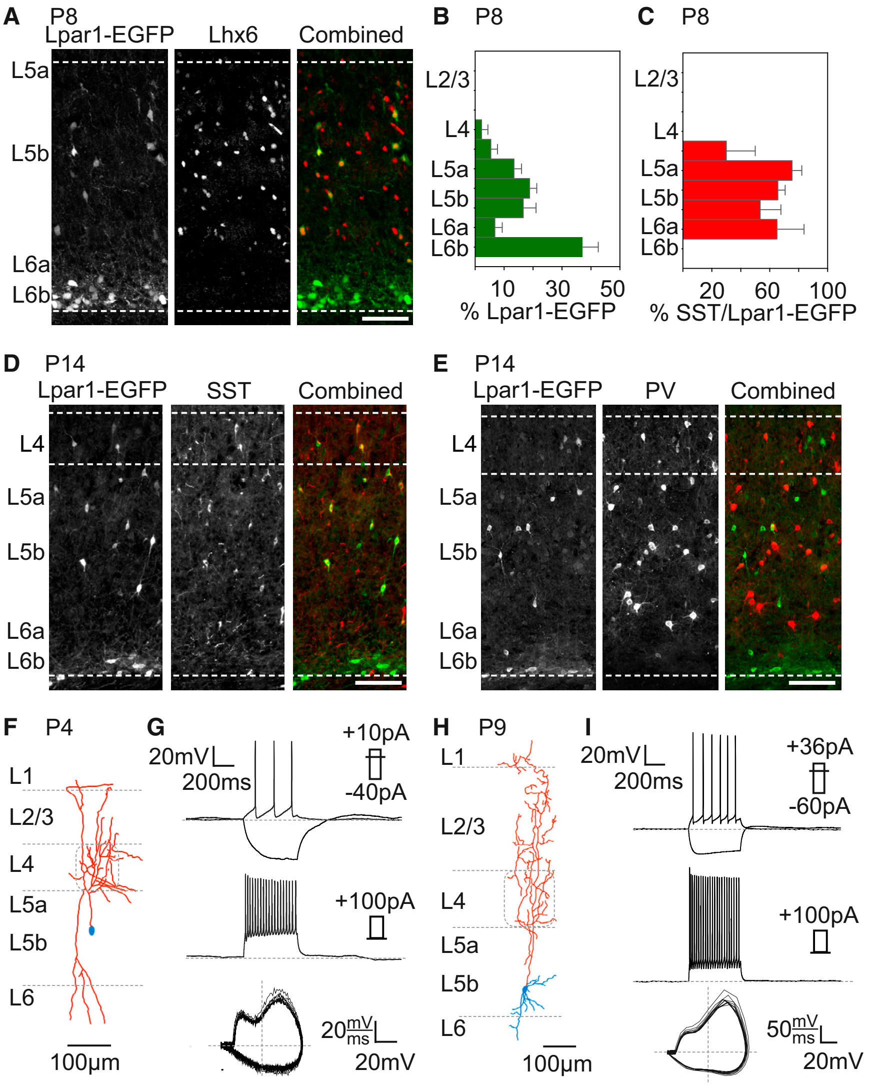

Figure 1. Lpar1-EGFP Labels a Population of Infragranular SST+, Non-Fast Spiking Martinotti Cells

(A) The *Lpar1-EGFP* transgene labeled cells (left panel) in P8 S1BF layer (L)5b that expressed Lhx6 (middle) in contrast to the other population of GFP+ cells in L6b (subplate) that are Lhx6-negative (right). y axis, location of the cortical layer (layer 5a to 6b); top dashed white line, L4-L5a border; scale bar, 100 μm.

(B) The distribution of Lpar1-EGFP cells across the depth of a cortical column in P8 S1BF (n = 8 animals); y axis, average location of the cortical layer (L2/3 to 6b); error bars, ± SEM.

(C) The percentage of Lpar1-EGFP cells that expressed SST across the depth of the cortex at P8; data presented as in (B).

(D and E) At P15, L5b Lpar1-EGFP cells expressed SST (D) (n = 4 animals), whereas (E) none expressed the other principal marker of Lhx6+ INs, parvalbumin (PV); scale bar, 100 μm.

(F) Reconstruction of an early (P4) biocytin-filled L5b Lpar1-IN. Even at early ages, the axon (red) of L5b Lpar1-INs extended to L1, characteristic of Martinotti cells.

(G) Intrinsic electrophysiological profile of the Lpar1-IN shown in (F); top traces, current clamp response to threshold and hyperpolarising current step injections which identified the cell as a low-threshold spiking IN. Middle trace, response to depolarizing current injection to near-maximal firing frequency revealed spike frequency adaptation characteristic of non-fast spiking (NFS) INs. Bottom trace, AP phase (*dV/dt*) plot with a biphasic component during the rising phase of the AP typical of NFS subtypes regardless of developmental age.

(H and I) Corresponding data for a P9 Lpar1-IN exhibiting extensive axonal arborisation (red) in layers 4 and 2/3 (H). The intrinsic electrophysiological profile of the P9 Lpar1-IN was consistent with a low-threshold, adapting NFS subtype (I).

Lpar1-INs Receive Transient Early Excitatory Inputs from L4

We next examined the synaptic integration of L5b Lpar1-INs into the neocortical glutamatergic network. We performed laser-scanning photostimulation (LSPS) of caged glutamate—calibrated to the developmental age (Anastasiades and Butt, 2012)—to map the source of afferent input from glutamatergic neurons across the immediate S1BF cortical column through the first 2 postnatal weeks (Figure 3). The sum of the laser-evoked EPSCs in recorded Lpar1-INs revealed an increase in the total columnar excitatory input toward the end of the first postnatal week that then remained constant through the second week (Figure 3A). Analysis of the LSPS data revealed a transient, early (<P10), translaminar source of excitatory input onto Lpar1-INs that originated from L4 (Figure 3B) and was absent following the end of the L4 CPP (Figure 3B; P10–P15) (Crair and Malenka, 1995). Quantification of layer-specific input over development confirmed a decrease in input from L4 (Figures 3C and S1A), concomitant to an increase in that originating from L5b. No changes were observed in other layers (Figures 3C and S1A). Moreover, while this local input often extended into L6, there was little evidence for connections onto Lpar1-INs from either L6 corticothalamic PYRs or L6b SPNs at the earliest ages recorded (Figures 3B and 3D). Therefore, concurrent with the engagement of Lpar1-INs by thalamic afferent input, there is a gradual reorganization of local cortical excitatory input onto these cells such that the source of columnar input reconfigures from a translaminar L4 (Figure 3D; P4–P6) to a L5b-dominated motif (Figure 3D; P10–P15) (Figure S1A, right panel).

L4 SSNs Receive Transient Early GABAergic Inputs from L5b

Combined, these data suggest that L5b Lpar1-INs are well placed to exert GABAergic control over early TC signaling. L5 inhibition of more superficial layers, including L4, has been reported in mature neocortex (Buchanan et al., 2012; Kätzel et al., 2011) but has not been documented in the developing brain. To examine this possibility, we employed a modified

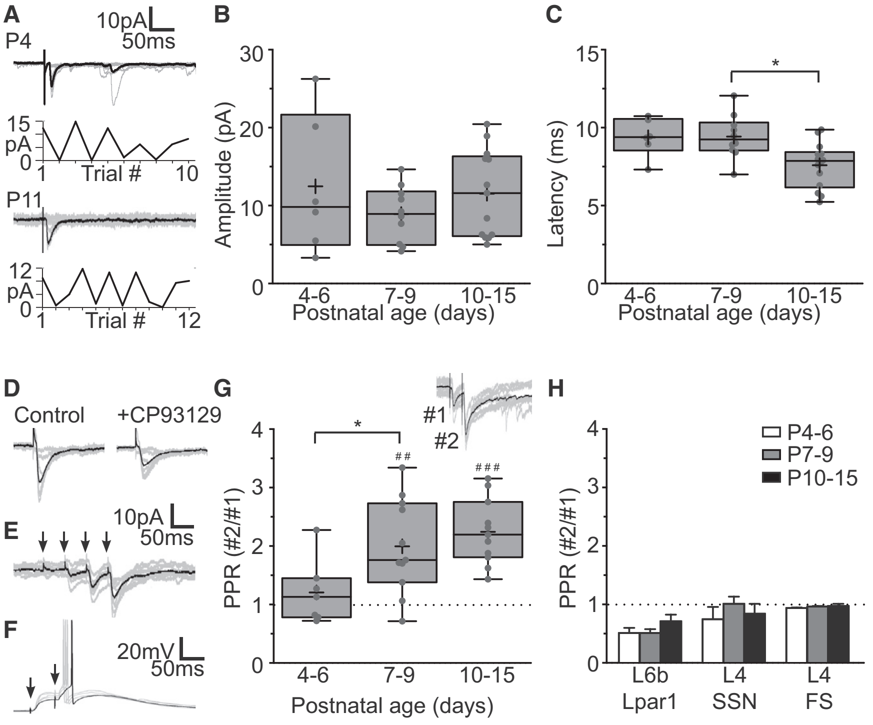

Figure 2. Maturation of Thalamic Input onto Lpar1-INs over Early Postnatal Development

(A) Voltage-clamp (VC; hp, -70 mV) recordings of synaptic responses (EPSC) observed in Lpar1-INs in response to electrical stimulation of the VPM at P4 (top panels; n = 10 sweeps, 60 s intervals) and P11 (bottom panels; n = 12 at 30 s intervals); corresponding plots, minimal electrical stimulation defined as when EPSCs were evoked on 50%–70% of trials.

(B) EPSC amplitude (pA) recorded in Lpar1-INs during minimal stimulation of the VPM P4–P6 (n = 8), P7–P9 (n = 8), and P10–P15 (n = 14). Boxplot, small gray circles depict average EPSC amplitude for each cell; horizontal line, median; cross, mean; box, standard deviation; error bars, the spread of the data.

(C) Latency to onset of the EPSC recorded in Lpar1-INs during minimal stimulation of the VPM. A difference was observed in the latency recorded in the P7–P9 and P10–P15 groups (*p = 0.009; Kruskal-Wallis (K-W) test, H(2,28) = 9.463; Dunn)

(D) Control TC-EPSCs (left) and those observed following 10 min perfusion with CP93129 (right).

(E) Response of Lpar1-INs to repeat electrical stimulation (20 Hz; minimal stimulation) of the VPM at P8 (hp, -70 mV).

(F) Suprathreshold response observed in an Lpar1-IN following paired-pulse stimulation (20 Hz) of the VPM at P11 under current clamp.

(G) Paired-pulse ratio (PPR) of TC-EPSCs in Lpar1-INs through early postnatal development; inset, example paired-pulse response (hp, -70 mV). For each cell (small gray circles) 10–20 stimulation sweeps were averaged; #, significant short-term plasticity (##p = 0.002 T[10] = 4.1; ###p < 0.001 T(10) = 7.4; one-sample t test); *, significant difference between groups (ANOVA p = 0.011, F(2,26) = 5.366).

(H) TC-EPSC PPR of *Lpar1-EGFP* L6b subplate neurons, L4 SSNs, and L4 Fast-Spiking (FS) INs at P4–P6 (light gray), P7–P9 (dark gray), and P10–P15 (black histogram bars); n $\geq$ 6 for each bar.

LSPS strategy using a caesium-based intracellular solution (Xu and Callaway, 2009) to assess the relative contribution of L5b INs to total columnar GABAergic input onto L4 SSNs.

GABAergic input onto SSNs was observed from P4–P6 and increased following the CPP (Figure 4A), in line with previous reports (Chittajallu and Isaac, 2010; Daw et al., 2007; Yang et al., 2013b). Our LSPS strategy confirmed that L5b was the dominant source of GABAergic input onto SSNs early in development (Figure 4B). However, similar to the L4 glutamatergic input onto Lpar1-INs (Figure 3B), this translaminar GABAergic input was transient and absent in later (P10–P15) recorded cells (Figure 4B). The layer source (Figure 4C) and relative distribution (Figure S1B) of GABAergic input underwent a reorganization over the time period studied such that after P9, GABAergic input originating from L5b decreased, whereas L4 input increased (Figure 4C). Comparison of average maps (Figure 4D) further highlights the transition from translaminar (L5b) to intralaminar (L4) GABAergic control of SSNs by the end of the CPP (An et al., 2012; Crair and Malenka, 1995; Isaac et al., 1997).

As such, the LSPS data reveal the existence of a transient developmental connection between L4 and L5b. This circuit is disassembled at the same time that sensory-dependent FS IN-mediated inhibition emerges within L4 barrels (Chittajallu and Isaac, 2010). This led us to speculate that, similar to the maturation of L4 FS to SSN synapses, disassembly of L5b GABAergic input onto SSNs is also dependent on normal sensory activity.

Lesioning of Sensory Afferents in the Periphery Arrests the Developmental Remodeling of L5b GABAergic Projections onto SSNs

To test the hypothesis that an intact sensory pathway is required for the switch in source of GABAergic input from L5b to L4, we transected the infraorbital nerve (ION) of mouse pups at P1. Although ION damage can lead to alterations beyond purely preventing transmission of sensory activity, we chose this method over whisker trimming or plucking, so as to completely eliminate the relay of passive early tactile experience from the periphery (Erzurumlu and Gaspar, 2012; Higashi et al., 1999). We then mapped GABAergic input onto SSNs in S1BF of the sensory-deprived (ION_{cut}) hemispheres during the first (Figure 5A) and second (Figure 5B) postnatal weeks of development. At P4–P6, total GABAergic input onto SSNs was reduced in ION_{cut} animals (Figure 5C) when compared to our previous data in which animals had not undergone surgical manipulation (Figure 4). However, this recovered to levels observed in controls by P10–P15 (Figure 5C). Despite the reduction observed in input at P4–P6, the normalized distribution exhibited a similar laminar organization between control and ION_{cut} animals (Figures 5A and 5D). However, at P10–P15 it differed between ION_{cut} and control recordings in that L5b input was preserved (Figures 5B and 5E), an observation not accounted for by changes in the intrinsic excitability of L4 and L5b INs (Table S2). These data suggest that intact, normal whisker-dependent sensory experience is

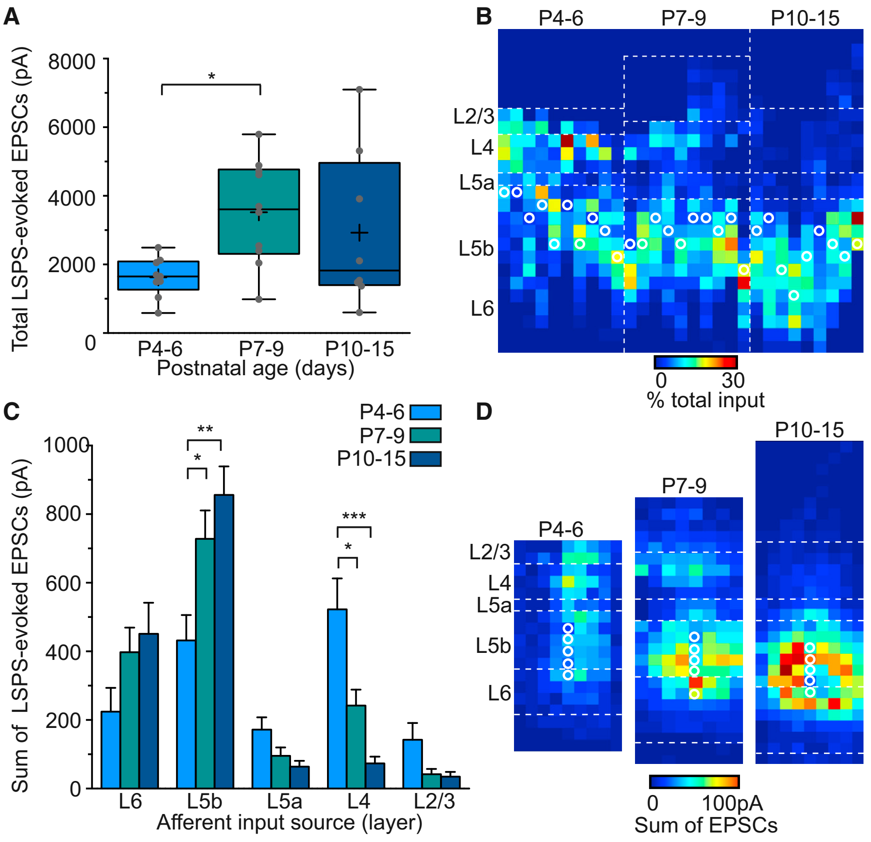

Figure 3. LSPS of Caged Glutamate Reveals a Developmental Rearrangement in the Laminar Organization of Excitatory Synaptic Inputs onto L5b Lpar1-INs

(A) Total LSPS-evoked excitatory synaptic input onto Lpar1-INs over early postnatal development; (*p = 0.046; ANOVA, F[2,24] = 2.771). Boxplots shown as in Figure 2.

(B) Excitatory inputs onto Lpar1-INs plotted across the depth of the cortex for all recorded cells (n = 29 cells). Each vertical array depicts the percent distribution of excitatory input onto a single recorded cell, the position of which is indicated by a white circle; dashed white lines, average layer boundaries. Cells are ordered by age, left to right, from P4 to P15.

(C) Laminar distribution of excitatory synaptic input onto Lpar1-INs over development. After P4–P6, L5b input increases (ANOVA: P7–P9, *p = 0.034, F[2,26] = 2.55; P10–P15, **p = 0.004, F(2,26) = 3.59, BfC), whereas L4 input decreases (ANOVA: P7–P9, *p = 0.039, F(2,26) = 2.67; P10–P15, $p < 0.001$, F(2,24) = 4.49, BfC).

(D) Average maps of excitatory synaptic input onto Lpar1-INs. Within each age group, maps of individual cells were aligned by the L4/5a border and input in each pixel averaged.

required for the transition from an early L5b to a late L4 GABAergic circuit impinging on SSNs. Furthermore, while local L4 GABAergic synaptic input onto SSNs shows an increase in a manner largely independent of our manipulation of sensory activity (Figure 5F), input from L5b was upregulated in ION_{cut} animals compared to controls (Figure 5G). Thus it appears that in the absence of appropriate whisker input, the L5b GABAergic projection onto SSNs can act to compensate for the delayed maturation of local, putative PV+ IN input (Daw et al., 2007), an observation not evident in the reciprocal excitatory input onto the L5b Lpar1-INs (Figure S2). This implies an intimate, "see-saw" relationship between innervation of SSNs by L5b and L4 INs (Takesian et al., 2013).

Conditional Silencing of SST+ INs Abolishes Early L5b GABAergic Input onto SSNs

Our data point to a role for L5b SST+ Lpar1-INs in early sensory integration in the neocortex at a time when PV+ INs are yet to be engaged by the thalamus. To confirm that the source of GABAergic signaling from L5b was indeed SST+ INs, we employed a conditional genetic silencing strategy to abolish AP-dependent synaptic vesicle release of GABA from INs targeted using *SST-ires-Cre* (Taniguchi et al., 2011) (Figure S3A). Our breeding paradigm resulted in the generation of pups that possessed SST neurons that were wild-type (WT; absence of Cre recombinase), conditional heterozygote (cHet; *SST-ires-Cre;Snap25*$^{C/+}$) or conditional knockout (cKO; *SST-ires-Cre;Snap25*$^{C/C}$) for the SNARE complex protein SNAP25 (Washbourne et al., 2002). We first confirmed that SST+ cells were present in early postnatal S1BF cortex (Figure S3B) of cKO animals. We then tested for the absence of SST+ IN signaling in cKO pups, by breeding onto the same background an optogenetic actuator that enables cell selective LSPS using conditional expression of the rat P2X2 receptor (see Anastasiades et al., 2016; Miesenböck, 2011; Zemelman et al., 2003) and focal UV laser uncaging of ATP, an approach that enables somatic localization of presynaptic INs in the developing neocortex (Figures S3C–S3F). LSPS ATP-evoked responses were recorded in SSNs from control (*SST-ires-Cre; rP2X2*) (Figure S3C) and cHet (Figure S3E) neonates. However, WT animals (Figure S3D) or cKO pups (Figure S3F) exhibited no response, which confirms the specificity of ATP uncaging and conditional silencing of SST+ INs following deletion of *Snap25*, respectively. Having established that SST+ cells were no longer capable of AP-dependent release of neurotransmitter in cKO pups, we next examined the proportion of L5b input that can be attributed to these INs using LSPS glutamate uncaging to map total GABAergic input onto SSNs (voltage clamped at E_{Glut}) in WT, cHet (data not shown), and cKO animals during the window when the L5b input is normally present. This revealed an absence of L5b GABAergic input onto SSNs in cKO animals (n = 6 cells recorded from 3 animals; WT, n = 5 cells, 4 animals) at early ages (P4–P6; Figure 6A) with a compensatory increase in local L4 GABAergic signaling at this age (Figure 6B). Concurrently, we tested for thalamic afferent input onto recorded SSNs in

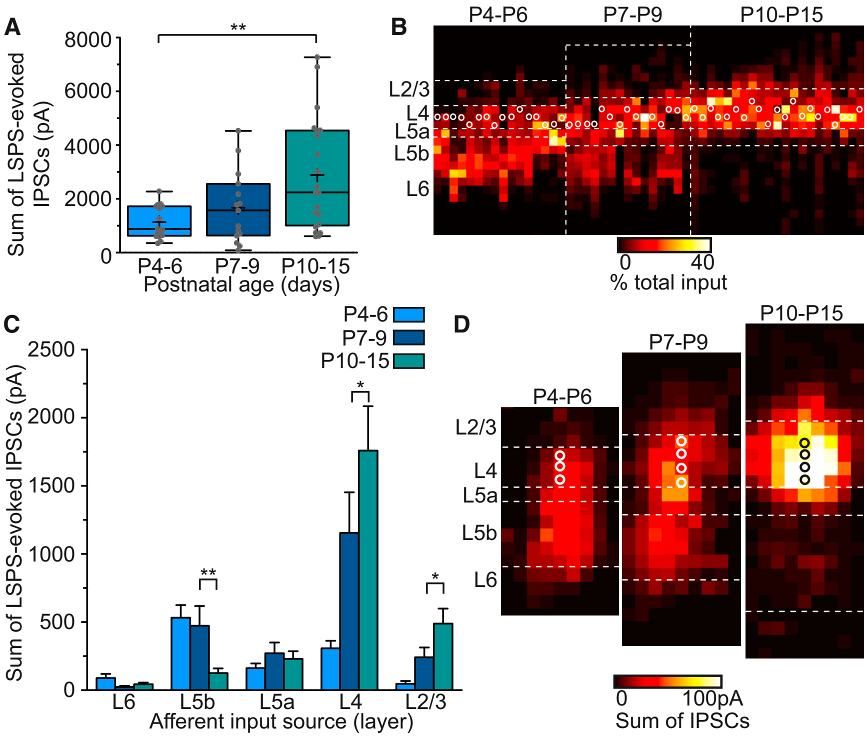

Figure 4. LSPS of Caged Glutamate Reveals a Developmental Rearrangement in the Spatial Organization of GABAergic Inputs onto S1BF L4 SSNs in the Early Postnatal Brain

(A) Total columnar GABAergic synaptic input onto L4 SSNs through development. Values correspond to the total sum amplitude of LSPS-evoked GABAergic inputs onto SSNs; boxplots as in Figure 2 (K-W test, **p = 0.016, H[2,49] = 8.25; Dunn).

(B) Remodeling of GABAergic inputs onto SSNs through development; plotted as for Figure 3B.

(C) Laminar organization of GABAergic input onto SSNs. Between P4–P6 and P10–P15, L5b input decreases (K-W test **p = 0.004, H[3,52] = 18.7; Dunn), while L4 and L2/3 input increases (L4, K-W test, *p = 0.011, H[2,49] = 23.0, Dunn; L2/3, Kruskal-Wallis test *p < 0.028, H[3,52] = 24.5, Dunn).

(D) Average maps of GABAergic synaptic input onto SSNs through early development; alignment as in Figure 3D.

these animals. In WT slices, we routinely recorded TC-EPSCs in SSNs (9/13 cells; n = 7 pups), yet no connectivity was observed in cKO (0/7 cells; n = 4 animals) (Figure 6D), even though TC innervation of the cortex had been confirmed by recording TC-ESPCs onto SPNs (Figures S3G and S3H). By P7–P9, GABAergic input onto SSNs had begun to collapse into the barrel in WT animals (Figure 6E; n = 6 cells, 4 animals) and had a similar distribution to that recorded from SSNs in cKO pups (n = 6 cells, 4 animals; Figures 6E and 6G), albeit there was reduced total GABAergic input onto SSNs in the latter (Figure 6F). At this age, TC-EPSCs could be evoked by electrical stimulation in all WT SSNs (6/6 cells; n = 4 animals) and half of those recorded in cKO animals (5/10 cells; n = 4 pups) (Figure 6H).

Our conditional silencing strategy confirms that SST+ INs provide early postnatal translaminar input onto SSNs. The data point to an interaction between translaminar and local inhibition of SSNs—similar to the ION transection experiments—and suggest that this early pathway might have a role to play in the timely acquisition of thalamic input by SSNs, with the caveat that our genetic strategy is not exclusive to the neocortex and may also influence signaling in the thalamus.

Molecular Determinants of the Layer 5b-4 Early Developmental Loop

Beyond activity, a number of molecular determinants have been shown to influence the formation of IN afferent and efferent synapses. Of these, the neuregulin 1 (Nrg1) receptor family has been shown to selectively regulate the formation of PV+ IN-pyramidal cell synaptic connections through ErbB4 signaling (Fazzari et al., 2010). We hypothesized that perturbation of Nrg1-ErbB4 signaling could indirectly influence the developmental relationship between translaminar L5b and local L4 GABAergic control and thereby further confirm the early developmental link between these pathways. To test this we took advantage of a transgenic mouse line that overexpresses Ig-Neuregulin-1$^{type\ 1}$ (Nrg1$^{type1\text{-}tg}$) in the cerebral cortex (Deakin et al., 2009; Deakin et al., 2012) during the CPP (Figure S4), with the objective of prematurely enhancing the local PV+ to SSN GABAergic microcircuit. To examine the impact of this genetic manipulation we first mapped GABAergic input onto L4 SSNs in Nrg1$^{type1\text{-}tg}$ pups (P4–P15). Throughout the time period studied, total GABAergic input onto SSNs was unchanged in Nrg1$^{type1\text{-}tg}$ transgenic (tg) animals when compared to age-matched WT littermates (Figure 7A), an observation that was mirrored in the amplitude and frequency of spontaneous synaptic activity recorded in SSNs and INs under both conditions (Figures S4B–S4E). The distribution of GABAergic input across the depth of the cortex—including the prominent early L5b input, was the same for WT animals (Figure 7B), as seen in controls (Figure 4B). In contrast, we never observed GABAergic input from L5b onto SSNs in tg animals, with SSNs recorded from tg animals only ever receiving local L4 GABAergic input (Figure 7C). Analysis of the distribution of input at P4–P6 revealed that there was a decrease in input from L5b INs in tg SSNs, compensated for by an increase in local synaptic input within L4 (Figure 7D). By P7–P9, the normal developmental increase in local L4 input observed in WT SSNs matched that recorded in tg littermates (Figure 7E). Some L5b input was present onto WT SSNs at this age but absent from cells recorded from tg animals (Figure 7E). By P10–P15 the laminar profiles of GABAergic input in tg and WT animals were indistinguishable, with the majority of GABAergic input impinging on SSNs originating from the immediate layer (Figure 7F).

Therefore, while total GABAergic input remains unchanged in tg compared to WT pups (Figure 7A), there was a significant

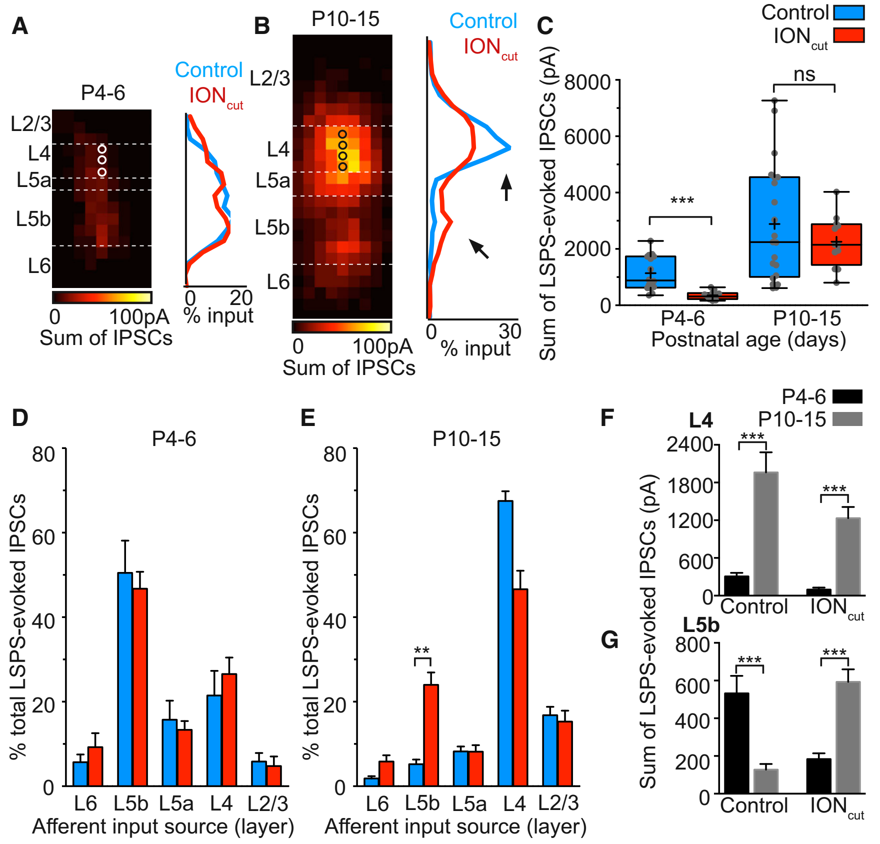

Figure 5. Sensory Perturbation as a Result of ION Transection Delays the Transition to a Local L4 GABAergic Circuit

(A) Average map (left) of evoked GABAergic input onto L4 SSNs in ION transected (ION$_{cut}$) animals at P4–P6. Right, normalized laminar profile of GABAergic input onto SSNs recorded from control (blue) and ION$_{cut}$ (red) animals.

(B) Corresponding data for SSNs in ION$_{cut}$ animals at P10–P15 (left). The normalized laminar profile (right) revealed an increase in L5b and a decrease in L4 GABAergic synaptic input (arrows) in ION$_{cut}$ animals (red) as compared to control (blue).

(C) GABAergic input onto SSNs at P4–P6 is significantly reduced (***$p < 0.001$, U = 10, M-W U test) compared to control, but recovers by P10–P15 (not significant [ns], p = 0.685 U = 115, M-W test).

(D and E) Normalized laminar GABAergic input onto SSNs showed no difference between ION$_{cut}$ and control at P4–P6 (D), but an increase in input from L5b in ION$_{cut}$ (n = 12) compared to controls (n = 21) at P10–P15 (E) (L5b, **p = 0.006 K-W test, H[1,45] = 124.9; Dunn); error bars, ± SEM.

(F) Total intralaminar (L4) GABAergic input onto SSNs between P4–P6 (black bars) and P10–P15 (gray) showed an increase in both control and ION$_{cut}$ (control, ***$p < 0.001$ U = 19; ION$_{cut}$, ***$p < 0.001$ U = 0, M-W test); error bars, ± SEM.

(G) Plot of total translaminar (L5b) GABAergic input onto SSNs between P4–P6 and P10–P15 showed a decrease in control but an increase in ION$_{cut}$ (control, ***$p < 0.001$, U = 30; ION$_{cut}$, ***$p < 0.001$, U = 3, M-W test); error bars, ± SEM.

decrease in L5b input (Figure 7D) in the former. This suggests that genetic perturbation of local GABAergic innervation of L4 SSNs occurs at the expense of L5b innervation at early ages (P4–P6; Figure 7D) and further suggests that the timing of the transition from the transient to mature circuit configuration is in part controlled by a need to maintain the appropriate level of GABAergic control over SSNs.

Molecular Determinants of Thalamocortical Integration onto SSNs

The convergence of early thalamic and cortical glutamatergic afferent input onto the L5b SST+ INs suggests that this population of IN might play a hitherto unappreciated role in thalamocortical synaptic integration. The absence of early (P4–P6) GABAergic transmission from L5b onto SSNs in the Nrg1$^{type1\text{-}tg}$ animal (Figures 7C and 7D) provided us with a means to test this idea without directly affecting synaptic connections within the thalamus. We performed TC stimulation using the same paradigm as for controls (Figure 2) in WT and tg littermates. Using electrical stimulation, we could readily evoke TC-EPSCs onto SSNs in WT animals at both early and late time points (Figure 7J and 7K). However, we were unable to evoke TC-EPSCs onto recorded SSNs in tg slices at early ages (n = 16 cells). At later ages there was a partial recovery, with TC-EPSCs observed post-CPP (Figure 7J). However, the amplitude of the EPSCs was significantly smaller in SSNs recorded in tg compared to WT animals (Figure 7K). To discount that this was due to delayed TC innervation in tg animals, we switched to recording L5b Lpar1-INs using *Lpar1-EGFP;Nrg1*$^{type1\text{-}tg}$ double transgenic animals. We observed no difference in either the amplitude (Figure 7L) or latency (data not shown) of TC-EPSCs evoked in Lpar1-INs in WT and tg animals, which suggest a specific failure of the thalamocortical synapse onto SSNs. These data indicate that the transient L5b-L4 circuit has a role to play in the timely acquisition of TC synapses onto SSNs in S1BF.

DISCUSSION

Our experiments reveal a transient early reciprocal connection between thalamo-recipient SST+ Lpar1-INs in L5b and SSNs in L4, which is present prior to the emergence of local FS IN-mediated feedforward inhibition (Figure 8A). This places L5b SST+ INs in an ideal position to regulate early thalamic input onto L4. We show that formation and disassembly of the transient L5b-L4 circuit are sensitive to sensory, activity-dependent, and molecular cues. The absence of appropriate sensory activity at the onset of the CPP results in the failure of the L5b-L4 circuit to transition from an infragranular-dominated translaminar mode (P4–P9) to the local L4-dominated intralaminar configuration (P10+) (Figure 8B). We confirm that the translaminar pathway is exclusively mediated by SST+ INs at these early ages by conditionally silencing this population to abolish L5b GABAergic input (Figure 8C). Conversely, perturbing molecular cues toward promoting FS IN integration biases the early (P4–P6) circuit toward

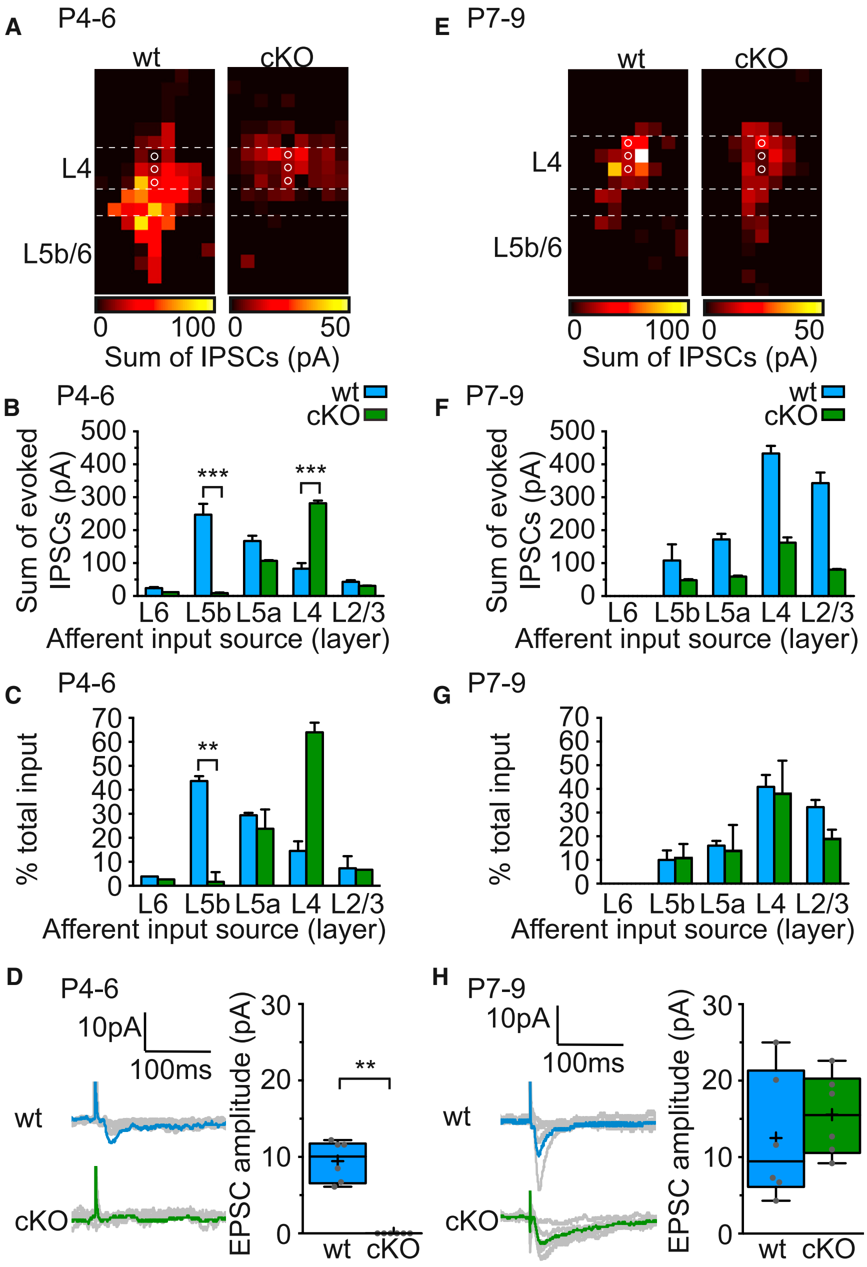

Figure 6. Conditional Knockout of Snap25 in SST+ INs Removes Early L5b GABAergic Input and Alters the Timeline for the Acquisition of L4 TC-EPSCs

(A) Average LSPS map of GABAergic input onto early (P4–P6) L4 SSNs in wild-type (WT; n = 5 cells; 4 animals) and conditional knockout (cKO; *SST-ires-Cre; Snap25*$^{C/C}$ mice; n = 6 cells; 4 animals).

(B) Laminar distribution of GABAergic input onto SSNs reveals a decrease in input from L5b (***$p < 0.001$, ANOVA $F[12,95] = 9.259$) but an in increase in local L4 input (***$p < 0.001$, ANOVA $F[12,95] = 7.742$); error bars, ± SEM.

(C) Normalized distribution of GABAergic input onto SSNs (L5b, **$p = 0.002$; K-W, $H[10,55] = 49.01$; Dunn test); error bars, ± SEM.

(D) Left, TC-EPSCs in SSNs from WT and cKO animals in which TC connectivity to cortex had been confirmed in SPNs. Right, TC-EPSC amplitude in WT (blue box) and cKO (green) pups; gray circles, average TC-EPSC amplitude for each cell; **$p = 0.002$, M-W test.

(E–H) Corresponding data for SSNs recorded from P7–P9 WT (n = 6 cells) and cKO (n = 6) animals.

a local GABAergic configuration at the expense of the L5b route (Figure 8D). In the absence of L5b GABAergic signaling, we observe a delay in thalamic afferent connectivity onto SSNs (Figures 8C and 8D). Together, these data suggest that the early L5b SST pathway onto L4 SSNs is a determinant of the time course for normal thalamic engagement and maturation of L4 function.

An Early, Transient Translaminar GABAergic Projection

Translaminar GABAergic projections have been previously described in mature motor, visual, and somatosensory cortices (Bortone et al., 2014; Buchanan et al., 2012; Kätzel and Miesenböck, 2014; Kätzel et al., 2011). These reports suggest that such inhibitory motifs could be modality specific, reflecting functional specializations of different areas (Kätzel et al., 2011)—for example, deep-layer GABAergic projections onto L4 neurons in adult V1 (Kätzel et al., 2011; Bortone et al., 2014) are absent in S1 (Kätzel et al., 2011; Figure 4). Our data, however, confirm the presence of such a translaminar connection in early S1BF development. Moreover, in contrast to visual cortex where translaminar inhibition has been shown to be mediated by FS, PV+ basket cells (Buchanan et al., 2012; Bortone et al., 2014), the transient L5b connection we describe in developing S1BF, emanates from NFS, SST+ INs, which are co-labeled by the *Lpar1-EGFP* transgene over the time periods examined. It remains to be seen why this translaminar GABAergic connection should be subserved by different IN types in S1 and V1. Moreover, why this connection is present in adulthood for one modality (V1), but eliminated post-CPP in the other (S1BF), is unknown. One possibility is that these differences are due to distinct sensory processing and computational requirements of the various modalities in response to differing thalamic engagement (Pouchelon et al., 2014), but further experimentation will be needed to establish this.

Thalamocortical Integration in Neonatal Cortex

Our data identify a transient, translaminar GABAergic circuit at the heart of the thalamocortical network in mouse S1BF, one that precedes the emergence of local L4 GABAergic circuits. This adds to the literature detailing a variety of mechanisms

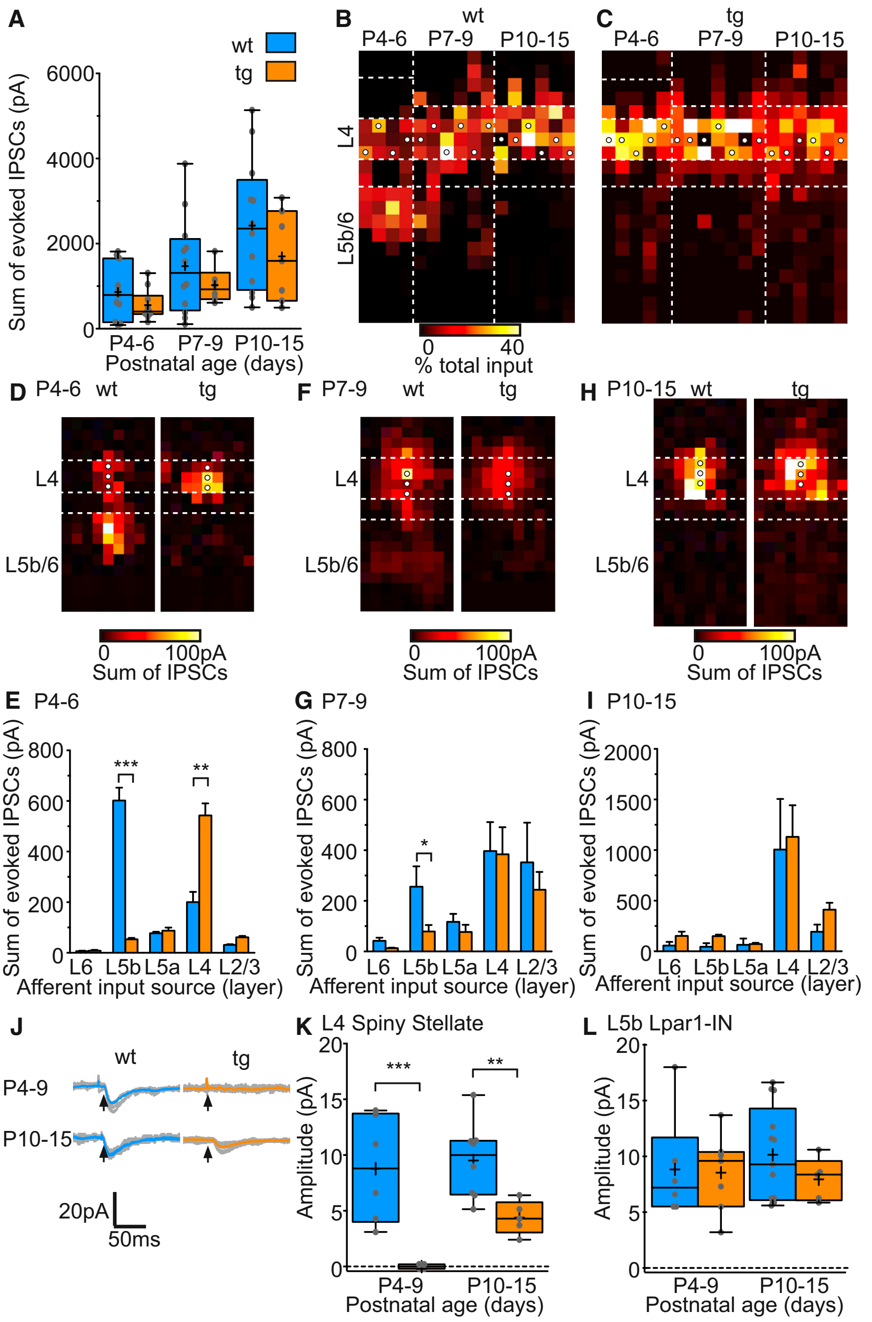

Figure 7. Failure of Early L5b GABAergic Synaptic Signaling and Delayed TC Input onto SSNs in an Nrg1^{type1}-Overexpressing Mouse Line, Nrg1$^{type1-tg}$

(A) Total GABAergic input onto L4 SSNs in Nrg1$^{type1-tg}$ tg and nontransgenic WT littermates through early development.

(B and C) The relative distribution of GABAergic inputs onto SSNs across the depth of the cortex in WT and tg animals; plots formatted as for Figure 3B.

(D) Top: average maps of GABAergic synaptic input onto SSNs in WT and tg animals at P4–P6.

(E) Plot of the total laminar GABAergic input onto SSNs in WT and tg animals at P4–P6. (L5b, ***p < 0.001 U = 21; L4: **p = 0.010 U = 21, M-W test).

(F and G) Data for SSNs recorded at P7–P9 (L5b, **p = 0.022 U = 0, M-W test).

(H and I) Corresponding data for SSNs recorded at P10–P15.

(J) Voltage-clamp (hp, −70 mV) responses recorded in SSNs in response to thalamic stimulation at P4–P9 (top) and P10–P15 (bottom) in cells recorded from WT (blue) and tg (orange) animals. Individual sweeps (n = 10) shown in gray, average response in color; arrows, time of stimulus.

(K) Minimal stimulation TC-EPSC amplitude recorded in SSNs at P4–P9 (WT, n = 6; tg, n = 12 cells) and P10–P15 (WT, n = 5; tg, n = 8); blue bars, WT; orange, tg data (P4–P9, ***p < 0.001 U = 0; P10–P15, **p = 0.0062 U = 2, M-W test)

(L) Amplitude of TC-EPSCs recorded in L5b Lpar1-INs shown as in (K).

that control this critical juncture in cortical maturation (e.g., Chittajallu and Isaac, 2010; Crair and Malenka, 1995; Daw et al., 2007; Isaac et al., 1997; Minlebaev et al., 2011; Yang et al., 2013a) and further highlights the investment made by the developing brain in transient synaptic networks to direct circuit formation (e.g., Dupont et al., 2006; Kanold et al., 2003; Tolner et al., 2012). Such connections may not simply reflect developmental exuberance but rather constitute specialized devices that respond to the specific challenges of neurodevelopment, similar to early SPN circuits (Dupont et al., 2006; Kanold et al., 2003; Kanold and Luhmann, 2010).

The development of the somatosensory thalamocortical slice preparation (Agmon and Connors, 1991) allowed investigation of the synaptic mechanisms of thalamocortical developmental plasticity and maturation. Succinctly, it was found that glutamatergic thalamic inputs to L4 SSNs are one of the fundamental loci for plasticity between P3 and P8, and that the cellular mechanism for this process is long-term potentiation (LTP) via "unsilencing" of NMDAR-containing synapses via AMPAR insertion (Crair and Malenka, 1995; Isaac et al., 1997), a caveat being that such experiments were mostly conducted in the presence of GABA receptor antagonists and thus not poised to interrogate the contribution of early GABAergic circuit similar to that reported here. Recent work in vivo has reported a corresponding time window for LTP at the TC synapse with L4 (An et al., 2012). This links well with a number of experiments examining population activity in the developing barrel cortex in vivo, which have established EGOs as a network mechanism capable of potentiating TC inputs via multiple replay of correlated thalamic afferent activity and spiking in L4 (Minlebaev et al., 2011; Yang et al., 2013a). The circuit we report is well placed to control such early network activity and could bridge a conceptual gap

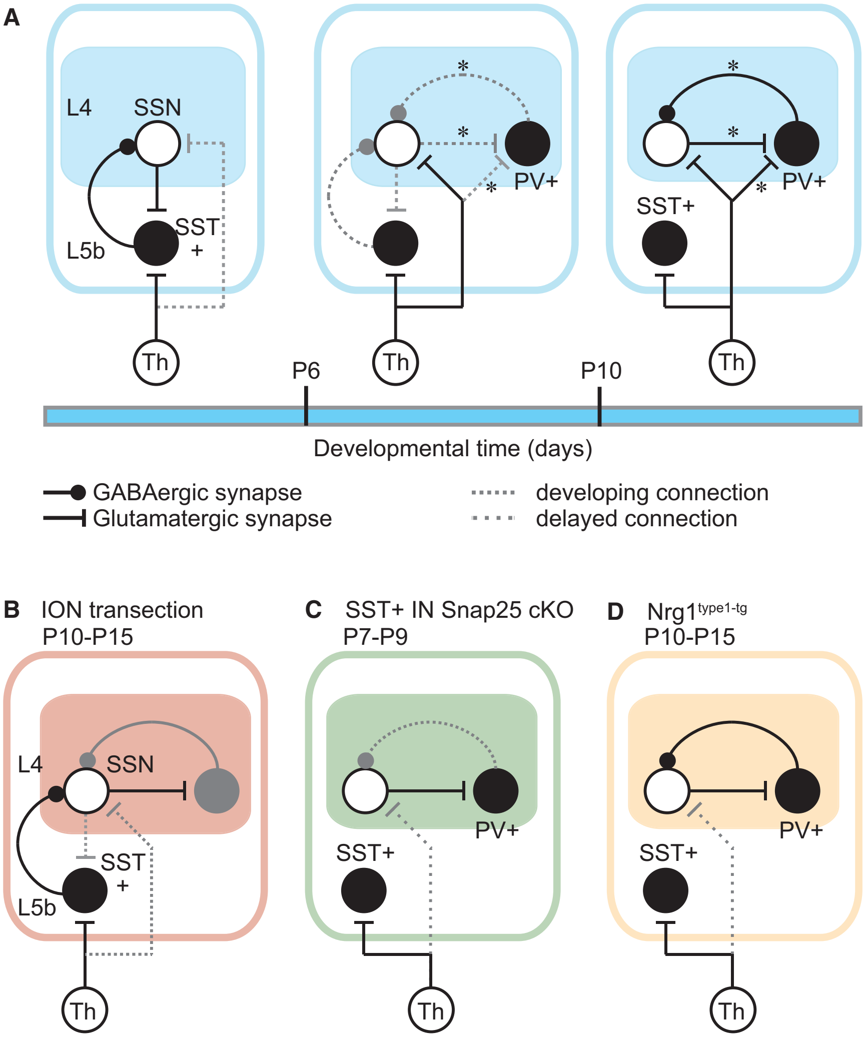

Figure 8. Transient Circuits Involving L5b SST+ GABAergic INs in the Early Postnatal S1BF

(A) Diagrams of the circuits revealed in the current study at early ages (left panel), toward the end of the CPP (middle) and post-CPP (right). Black circle, GABAergic IN; filled circle ending, GABAergic synapse; white circle, glutamatergic neuron; flat line ending, glutamatergic synapse; gray dotted line connector, connection undergoing remodelling. L4, layer 4; L5b, layer 5b; SSN, spiny stellate neuron; SST+, Lpar1-EGFP, SST-expressing IN; Th, VPM nucleus. PV+, parvalbumin-expressing IN; *, connections previously reported in the literature.

(B) Alterations to the post-CPP circuit observed following ION transection.

(C) Connections onto SSNs during the CPP following SST+ IN silencing by conditional knockout (cKO) of Snap25. Sparse dashed gray connector, a synaptic connection that is delayed relative to that observed in WT animals (see A).

(D) The early transient circuit in Nrg1$^{type1\text{-}tg}$ animals.

between the cellular-synaptic (Crair and Malenka, 1995; Isaac et al., 1997) and network (Minlebaev et al., 2011; Yang et al., 2013a) levels of analysis of TC synapse maturation. The recovery of thalamic input onto SSNs following all our manipulations supports the idea that multiple parallel TC pathways exist in the early postnatal brain (Luhmann et al., 2014), and suggests additional complexity in the way that these circuits combine to ensure appropriate circuit maturation.

Activity and the Maturation of GABAergic Circuits

Our data are in line with evidence that identify sensory experience and activity as critical determinants of L4 inhibitory circuit maturation (Chattopadhyaya et al., 2004; Chittajallu and Isaac, 2010; Jiao et al., 2006; Pouchelon et al., 2014; Sadaka et al., 2003; Sugiyama et al., 2008). Deficits in early activity or sensory experience impair proliferation and maturation of GABAergic synaptic contacts with SSNs, with a more pronounced effect reported for INs making contacts on the somatic compartment (Chattopadhyaya et al., 2004; Elgazzar et al., 2008; Jiao et al., 2006; Sadaka et al., 2003; Xue et al., 2014), which originate primarily from FS, PV+ basket cells. Using LSPS we reveal a compensation in the early GABAergic circuit under altered sensory drive: maintenance of infragranular synaptic input on SSNs to adjust for reduced local, putative PV-mediated inhibition (Daw et al., 2007; Xu et al., 2013), a reciprocal interaction that has been reported for auditory cortex (Takesian et al., 2010, 2013) and cell transplantation experiments (Tang et al., 2014). Taken together, this suggests an intimate, antagonistic relationship between these two IN classes and their synaptic pathways in the developing brain; biasing connectivity in favor of one results in a reciprocal alteration of the other. The only exception to this was observed at later time points following conditionally silencing of SST+ neurons, which supports a role for this pathway in the maturation of PV+ INs (see Tuncdemir et al., 2016, in this issue of *Neuron*). Under normal developmental circumstances, the translaminar SST+ pathway dominates early in development and via a sensory experience-dependent mechanism gives way to the local PV+ configuration.

It is unclear at present what the molecular and structural underpinnings of the translaminar to intralaminar transition in GABAergic signaling could be. Our data point to the importance of activity in determining the onset of the developmental remodeling. This is in line with a considerable body of evidence detailing the maturation of various glutamatergic cell types during these first few postnatal weeks, all of which undergo changes in somatodendritic morphology (Callaway and Borrell, 2011; Kasper et al., 1994; Koester and O'Leary, 1992; Piñon et al., 2009). The existence of similar mechanisms in INs has gained traction recently with the identification of activity-dependent transcription pathways that influence IN morphology and

developing GABAergic circuits (Bloodgood et al., 2013; Close et al., 2012; Donato et al., 2013; Spiegel et al., 2014; Xue et al., 2014). *Npas4*, for example, has been shown to be expressed by and regulate PYR and SST+ IN synaptic interactions (Bloodgood et al., 2013; Spiegel et al., 2014). In PYRs it promotes a redistribution of inhibitory synapses favoring the soma and decreasing dendritic inhibition (Bloodgood et al., 2013), whereas in SST+ INs, *Npas4* leads to an increase in afferent excitatory connectivity (Spiegel et al., 2014). *Satb1*, another such transcription factor, also regulates circuit formation in SST+ INs. Conditional deletion of *Satb1* in SST+ INs results in these cells receiving significantly less excitatory input than wild-type SST+ INs and compromised the efferent targets of these INs, with PYRs showing reduced inhibition as a result (Close et al., 2012). This suggests that transcriptional programs present in INs are ideally placed to interpret network activity and, as a result, trigger transitions in circuit organization such as the critical period plasticity.

EXPERIMENTAL PROCEDURES

Mouse Lines

Animal experiments were approved by the University of Oxford local ethical review committee and conducted in accordance with Home Office personal and project (70/6767; 30/3052; 30/2919) licenses under the UK Animals (Scientific Procedures) 1986 Act. The following mouse lines maintained on outbred (CD1/NIHS) backgrounds were used: *Lpar1-EGFP* [Tg(Lpar1-EGFP)GX193Gsat], *Nkx2-1Cre* [Tg(Nkx2-1-cre)Kess], *Z/EG* [Tg(CAG-Bgeo/GFP)21Lbe], *R26::P2X2R-EGFP* (floxed-stop-rat P2X2 receptor), *floxed-Snap25* [B6-Snap25tm3mcw], and $Nrg1^{tg\text{-}type1}$ mice (Michailov et al., 2004). All experiments were performed blind to the genotype, which was ascertained by PCR following completion of the data analysis.

In Vitro Slice Preparation

Mice of either sex (P3–P15) were deeply anesthetized with 4% isoflurane in 100% O_2 before decapitation and dissection of the brain in ice-cold, artificial cerebral spinal fluid (ACSF: 125 mM NaCl, 2.5 mM KCl, 25 mM $NaHCO_3$, 1.25 mM NaH_2PO_4, 1 mM $MgCl_2$, 2 mM $CaCl_2$, 20 mM glucose; pH equilibrated with 95% O_2/5% CO_2; all chemicals from Sigma unless otherwise specified). S1BF TC slices (400–500 μm) were cut in ice-cold ACSF using a vibratome. Slices were prepared according to established procedures (Agmon and Connors, 1991) and allowed to recover in ACSF maintained at room temperature (RT) for ~60 min prior to the onset of recording.

Whole-Cell Patch-Clamp Electrophysiology

Neurons were selected typically >50 μm below the slice surface. Cortical layers in acute in vitro slices were distinguished in the IR-DIC image based on cell size and density. The L4/5a boundary was identified via an abrupt transition from small spherical, densely packed cells to large, pyramidal-shaped sparsely distributed cells in L5a. The L5a/b boundary was apparent through an increase in cell density and the L5b/6a boundary through a decrease in cell size. Under low-magnification IR-DIC imaging in vitro, L5b could be observed as a distinct dark band. L6b could be distinguished from L6a by its diversity of somatic morphologies and horizontal orientation. Whole-cell patch-clamp recordings were obtained at RT using borosilicate glass microelectrodes (Harvard Apparatus, UK) of 6–9 MΩ resistance, forged using a PC-10 puller (Narishige, Japan). Electrodes were filled with either a potassium-based (128 mM K-gluconate, 4 mM NaCl, 0.3 mM Li-GTP, 5 mM Mg-ATP, 0.0001 mM $CaCl_2$, 10 mM HEPES, and 1 mM glucose) or Cs-based internal solution (100 mM gluconic acid, 0.2 mM EGTA, 5 mM MgCl, 40 mM HEPES, 2 mM Mg-ATP, 0.3 mM Li-GTP, ~7.3 pH using CsOH). Biocytin (~0.3%) was included to allow morphological reconstruction of the recorded cells. EPSCs were recorded in voltage clamp at −70 mV holding potential (hp). IPSCs were recorded by voltage clamping the cells at the equilibrium potential for glutamate (E_{glut}). E_{glut} was determined empirically by uncaging glutamate proximal to the recorded cell soma and adjusting the hp until no net current was observed.

Electrical Stimulation of Thalamic Afferents

To test for TC afferent input, a bipolar microelectrode (Harvard Apparatus, UK) was placed in the ventrobasal nucleus (VB) of the thalamus or the internal capsule (IC) and connected to a current isolator (DS3, Digitimer Ltd, UK). Stimulation strength (μA) was varied to evoke all-or-none, threshold postsynaptic responses observed at −70 mV according to a minimal stimulation protocol (Isaac et al., 1997; Raastad et al., 1992) with pulses of 200 μs duration delivered at interstimulus intervals of either 30 or 60 s depending on age. To find minimal amplitude responses, stimulation strength was adjusted until events of consistent latency and amplitude were evoked on 50%–70% of trials. TC-EPSCs were deemed monosynaptic if trial-to-trial latency variability was <2 ms and amplitude was consistent across trials. We defined absence of thalamic input to a cell as a failure to evoke TC-EPSCs with such characteristics regardless of stimulation amplitude having successfully recorded TC-EPSCs in cells located in the same barrel column known to receive input at that developmental time point (e.g., SPNs). PPR was investigated by recording for each cell 20 trials of VPM stimulation under minimal stimulation conditions. Each trial consisted of two 50 ms-spaced electrical pulses (20 Hz). PPR was calculated as the ratio between the amplitude (in pA) of the second and first TC-EPSC.

Laser-Scanning Photostimulation

LSPS was performed as previously described (Anastasiades and Butt, 2012). Laser target spots were organized in a grid with constant width of 450 μm but varying length according to developmental age (650–1,450 μm). Prior to photostimulation, slices were preincubated for a minimum of 6 min with high divalent cation (HDC) ACSF, which was identical in composition to normal ACSF but with raised levels (4 mM) of $MgCl_2$ and $CaCl_2$, and 100 μM MNI-caged glutamate (Tocris Bioscience, UK). Laser power was calibrated to the appropriate developmental age by mapping presynaptic neurons (PYRs or INs) in current clamp mode across the extent of grid, and then adjusting the power to restrict laser-evoked AP firing to the immediate 50 μm target spot directly over the cell soma, yet sufficient to elicit ~3 APs. This ensured a spatial resolution of ~50 μm in input maps regardless of developmental age. Putative monosynaptic event detection windows were defined as previously published (Anastasiades and Butt, 2012). Repeat runs were obtained for each LSPS grid. Current traces were analyzed offline with Minianalysis 6.0 (Synaptosoft Inc.), using the multipeak extrapolation function for summating PSCs. The number and amplitude of putative monosynaptic PSCs were extracted using a customized Matlab script (MathWorks). The sum amplitude of PSCs for each laser target spot (pixel) was calculated per run, and then averaged across all runs. A photomicrograph was taken of the targeting grid relative to the acute in vitro slice to enable reconstruction of the target points relative to the cortical layer boundaries. Total afferent synaptic input onto any given cell was calculated by summing the amplitude of average evoked PSCs across the extent of the grid. Vertical (layer) input profiles were computed by summing the synaptic input evoked from each 50 μm horizontal row and normalizing this value to the total synaptic input received by that cell. Laminar distribution of inputs was calculated to the nearest 50 μm pixel. Average maps were plotted aligned to L4-L5a border on the vertical axis with layers assigned according to the most frequent boundaries observed within any given age group.

Immunohistochemistry

Following terminal general anesthesia, mice were transcardially perfused with 4% PFA in PBS and postfixed for 1–2 hr depending on age. Brains were washed in PBS, cryoprotected by exposure to 10% then 30% sucrose in PBS before being embedded in O.C.T. (VWR) on dry ice. Tissues were cryosectioned at 14–16 μm and mounted on slides. Prior to immunohistochemistry, slides were washed with PBS, then PBST (0.1M PBS, 0.1% Triton X-100) and blocked with 2% donkey serum in PBST for 1 hr at RT. Slides were incubated with primary antibody (Ab) in blocking solution overnight at 4°C. The following Abs were used: rabbit anti-Lhx6 (1:400; gift from V Pachnis) rabbit anti-SST (AB5494, Millipore), rat anti-SST (MAB354, Millipore), mouse anti-PV

(MAB1572, Millipore), and chicken anti-GFP (ab13970, Abcam). Prior to incubation with the relevant secondary Ab (1:200; fluorophores: Cy2, Alexa488, Cy3, Alexa 546, Cy5, AMCA; Abcam/Millipore), slices were washed thoroughly in PBS for 2 hr at RT. Sections were washed and counterstained with DAPI before being mounted and sealed using nail polish. Images were acquired using a Zeiss laser scanning confocal microscope (LSM710).

Western Blot

P3/P8 mouse cortices were lysed in Pierce RIPA buffer (Thermo Scientific) containing Complete protease inhibitors (Roche) and analyzed for protein content using Bradford reagent. A total of 20 μg of protein extract was separated on a NuPAGE 3%–7% Tris-Acetate gel (Life Technologies) and blotted onto nitrocellulose. Immunoblotting was performed using Abs against Nrg1-type1 (1:1,000 dilution; ab27303, Abcam) and β-actin (1:500; ab8226, Abcam). Immunoreactive bands were detected by enhanced chemoluminescence (GE Healthcare).

Infraorbital Nerve Sectioning

P1 pups were anaesthetised on ice until they were unresponsive to tail or paw pinch. The skin on the left side of the face was wiped with Betadine (povidone iodine). A 1–2 mm skin incision was made at the ventral edge, just posterior to the whisker pad. The ION was lifted off the underlying blood vessel using forceps and cut through with an opthalmology scalpel. The skin edges were apposed but not sutured and the animal placed in the recovery chamber heated to 36°C. Once pups had recovered, they were returned to the dam. TC slices were then prepared as above to record from the contralateral (ION_{cut}) S1BF.

Morphological Reconstructions of Recorded Cells

Slices containing biocytin-filled cells were fixed in 4% PFA in PBS overnight at 4°C. After several PBS rinses, slices were then incubated in PBST for 1–2 hr. Then slices were transferred into 0.1% PBST containing Streptavidin-Alexa568 (1:500; Molecular Probes) and incubated overnight at 4°C. After several washes in PBS, slices were mounted and imaged. Confocal images of filled cells were selected for reconstruction using Fiji software (NIH).

Statistical Analysis

All statistical analysis was performed using Prism (Graphpad). Normality of the data was checked using the Shapiro-Wilk test. Differences in populations conforming to normality were tested using Student's t test or one-way ANOVA. In cases where normality assumptions were violated, Mann-Whitney (M-W), Kruskal-Wallis (K-W), and Wilcoxon tests were used. Bonferroni correction (BfC) and Dunn test (Dunn) were applied for multiple comparisons as appropriate. Alpha levels of $p \leq 0.05$ were considered significant. All means are presented ± SEM.

SUPPLEMENTAL INFORMATION

Supplemental Information includes four figures and two tables and can be found with this article at http://dx.doi.org/10.1016/j.neuron.2016.01.015.

AUTHOR CONTRIBUTIONS

A.M.-S., D.L., and S.J.B.B. designed the research, conducted experiments, analyzed the data, and wrote the manuscript. A.-K.K., J.A.S., and E.B.E.B. conducted experiments and analyzed the data. A.H.-S. conducted experiments and provided *Lpar1-EGFP* mice. M.C.W. provided the *floxed-Snap25* mouse line. Z.M. designed the research and provided the *Lpar1-EGFP* mouse line. All authors edited the manuscript.

ACKNOWLEDGMENTS

Research in the Butt lab was funded by the Medical Research Council (MR/K004387/1), the Brain & Behavior Research Foundation (Narsad; grant reference 19079), and the OUP John Fell Fund. Work conducted in the Molnár lab was supported by the MRC (G00900901). We would like to thank Drs. Kessaris (UCL, London) and Schwab and Nave (Max Planck Institute of Experimental Medicine, Göttingen) for mouse lines used in this study, Dr. Vassilis Pachnis for the gift of the Lhx6 antibody, and Anne Hedegaard (Oxford) for assistance with experiments. A.M.-S. and J.A.S. were supported by Wellcome Trust studentships (086362/Z/08/Z and 102386/Z/13/Z). A.M.-S. also received funding from FCT (Portugal). A.-K.K. was supported by a stipend from the German National Academic Foundation.

Received: February 5, 2015
Revised: August 28, 2015
Accepted: January 6, 2016
Published: February 3, 2016

REFERENCES

Agmon, A., and Connors, B.W. (1991). Thalamocortical responses of mouse somatosensory (barrel) cortex in vitro. Neuroscience *41*, 365–379.

Agmon, A., Hollrigel, G., and O'Dowd, D.K. (1996). Functional GABAergic synaptic connection in neonatal mouse barrel cortex. J. Neurosci. *16*, 4684–4695.

Allendoerfer, K.L., and Shatz, C.J. (1994). The subplate, a transient neocortical structure: its role in the development of connections between thalamus and cortex. Annu. Rev. Neurosci. *17*, 185–218.

An, S., Yang, J.W., Sun, H., Kilb, W., and Luhmann, H.J. (2012). Long-term potentiation in the neonatal rat barrel cortex in vivo. J. Neurosci. *32*, 9511–9516.

An, S., Kilb, W., and Luhmann, H.J. (2014). Sensory-evoked and spontaneous gamma and spindle bursts in neonatal rat motor cortex. J. Neurosci. *34*, 10870–10883.

Anastasiades, P.G., and Butt, S.J. (2012). A role for silent synapses in the development of the pathway from layer 2/3 to 5 pyramidal cells in the neocortex. J. Neurosci. *32*, 13085–13099.

Anastasiades, P.G., Marques-Smith, S., Lyngholm, D., Lickiss, T., Raffiq, S., Kätzel, D., Miesenböck, G., and Butt, S.J.B. (2016). GABAergic interneurons form transient, layer-specific circuits in early postnatal neocortex. Nat. Commun. *7*, http://dx.doi.org/10.1038/ncomms10584.

Ben-Ari, Y., Khalilov, I., Represa, A., and Gozlan, H. (2004). Interneurons set the tune of developing networks. Trends Neurosci. *27*, 422–427.

Bloodgood, B.L., Sharma, N., Browne, H.A., Trepman, A.Z., and Greenberg, M.E. (2013). The activity-dependent transcription factor NPAS4 regulates domain-specific inhibition. Nature *503*, 121–125.

Bortone, D.S., Olsen, S.R., and Scanziani, M. (2014). Translaminar inhibitory cells recruited by layer 6 corticothalamic neurons suppress visual cortex. Neuron *82*, 474–485.

Buchanan, K.A., Blackman, A.V., Moreau, A.W., Elgar, D., Costa, R.P., Lalanne, T., Tudor Jones, A.A., Oyrer, J., and Sjöström, P.J. (2012). Target-specific expression of presynaptic NMDA receptors in neocortical microcircuits. Neuron *75*, 451–466.

Butt, S.J., Fuccillo, M., Nery, S., Noctor, S., Kriegstein, A., Corbin, J.G., and Fishell, G. (2005). The temporal and spatial origins of cortical interneurons predict their physiological subtype. Neuron *48*, 591–604.

Callaway, E.M., and Borrell, V. (2011). Developmental sculpting of dendritic morphology of layer 4 neurons in visual cortex: influence of retinal input. J. Neurosci. *31*, 7456–7470.

Chattopadhyaya, B., Di Cristo, G., Higashiyama, H., Knott, G.W., Kuhlman, S.J., Welker, E., and Huang, Z.J. (2004). Experience and activity-dependent maturation of perisomatic GABAergic innervation in primary visual cortex during a postnatal critical period. J. Neurosci. *24*, 9598–9611.

Chittajallu, R., and Isaac, J.T. (2010). Emergence of cortical inhibition by coordinated sensory-driven plasticity at distinct synaptic loci. Nat. Neurosci. *13*, 1240–1248.

Chou, S.J., Babot, Z., Leingärtner, A., Studer, M., Nakagawa, Y., and O'Leary, D.D. (2013). Geniculocortical input drives genetic distinctions between primary and higher-order visual areas. Science *340*, 1239–1242.

Close, J., Xu, H., De Marco García, N., Batista-Brito, R., Rossignol, E., Rudy, B., and Fishell, G. (2012). Satb1 is an activity-modulated transcription factor required for the terminal differentiation and connectivity of medial ganglionic eminence-derived cortical interneurons. J. Neurosci. *32*, 17690–17705.

Crair, M.C., and Malenka, R.C. (1995). A critical period for long-term potentiation at thalamocortical synapses. Nature *375*, 325–328.

Crocker-Buque, A., Brown, S.M., Kind, P.C., Isaac, J.T., and Daw, M.I. (2015). Experience-dependent, layer-specific development of divergent thalamocortical connectivity. Cereb. Cortex *25*, 2255–2266.

Cruikshank, S.J., Urabe, H., Nurmikko, A.V., and Connors, B.W. (2010). Pathway-specific feedforward circuits between thalamus and neocortex revealed by selective optical stimulation of axons. Neuron *65*, 230–245.

Daw, M.I., Ashby, M.C., and Isaac, J.T. (2007). Coordinated developmental recruitment of latent fast spiking interneurons in layer IV barrel cortex. Nat. Neurosci. *10*, 453–461.

De la Rossa, A., Bellone, C., Golding, B., Vitali, I., Moss, J., Toni, N., Lüscher, C., and Jabaudon, D. (2013). In vivo reprogramming of circuit connectivity in postmitotic neocortical neurons. Nat. Neurosci. *16*, 193–200.

Deakin, I.H., Law, A.J., Oliver, P.L., Schwab, M.H., Nave, K.A., Harrison, P.J., and Bannerman, D.M. (2009). Behavioural characterization of neuregulin 1 type I overexpressing transgenic mice. Neuroreport *20*, 1523–1528.

Deakin, I.H., Nissen, W., Law, A.J., Lane, T., Kanso, R., Schwab, M.H., Nave, K.A., Lamsa, K.P., Paulsen, O., Bannerman, D.M., and Harrison, P.J. (2012). Transgenic overexpression of the type I isoform of neuregulin 1 affects working memory and hippocampal oscillations but not long-term potentiation. Cereb. Cortex *22*, 1520–1529.

Donato, F., Rompani, S.B., and Caroni, P. (2013). Parvalbumin-expressing basket-cell network plasticity induced by experience regulates adult learning. Nature *504*, 272–276.

Dupont, E., Hanganu, I.L., Kilb, W., Hirsch, S., and Luhmann, H.J. (2006). Rapid developmental switch in the mechanisms driving early cortical columnar networks. Nature *439*, 79–83.

Elgazzar, R.F., Mutabagani, M.A., Abdelaal, S.E., and Sadakah, A.A. (2008). Platelet rich plasma may enhance peripheral nerve regeneration after cyanoacrylate reanastomosis: a controlled blind study on rats. Int. J. Oral Maxillofac. Surg. *37*, 748–755.

Erzurumlu, R.S., and Gaspar, P. (2012). Development and critical period plasticity of the barrel cortex. Eur. J. Neurosci. *35*, 1540–1553.

Fazzari, P., Paternain, A.V., Valiente, M., Pla, R., Luján, R., Lloyd, K., Lerma, J., Marín, O., and Rico, B. (2010). Control of cortical GABA circuitry development by Nrg1 and ErbB4 signalling. Nature *464*, 1376–1380.

Friauf, E., McConnell, S.K., and Shatz, C.J. (1990). Functional synaptic circuits in the subplate during fetal and early postnatal development of cat visual cortex. J. Neurosci. *10*, 2601–2613.

Gil, Z., and Amitai, Y. (1996). Properties of convergent thalamocortical and intracortical synaptic potentials in single neurons of neocortex. J. Neurosci. *16*, 6567–6578.

Gil, Z., Connors, B.W., and Amitai, Y. (1999). Efficacy of thalamocortical and intracortical synaptic connections: quanta, innervation, and reliability. Neuron *23*, 385–397.

Hanganu, I.L., Kilb, W., and Luhmann, H.J. (2002). Functional synaptic projections onto subplate neurons in neonatal rat somatosensory cortex. J. Neurosci. *22*, 7165–7176.

Higashi, S., Crair, M.C., Kurotani, T., Inokawa, H., and Toyama, K. (1999). Altered spatial patterns of functional thalamocortical connections in the barrel cortex after neonatal infraorbital nerve cut revealed by optical recording. Neuroscience *91*, 439–452.

Higashi, S., Molnár, Z., Kurotani, T., and Toyama, K. (2002). Prenatal development of neural excitation in rat thalamocortical projections studied by optical recording. Neuroscience *115*, 1231–1246.

Hoerder-Suabedissen, A., and Molnár, Z. (2013). Molecular diversity of early-born subplate neurons. Cereb. Cortex *23*, 1473–1483.

Hubel, D.H., and Wiesel, T.N. (1964). Effects of monocular deprivation in kittens. Naunyn Schmiedebergs Arch. Exp. Pathol. Pharmakol. *248*, 492–497.

Hubel, D.H., and Wiesel, T.N. (1970). The period of susceptibility to the physiological effects of unilateral eye closure in kittens. J. Physiol. *206*, 419–436.

Isaac, J.T., Crair, M.C., Nicoll, R.A., and Malenka, R.C. (1997). Silent synapses during development of thalamocortical inputs. Neuron *18*, 269–280.

Jiao, Y., Zhang, C., Yanagawa, Y., and Sun, Q.Q. (2006). Major effects of sensory experiences on the neocortical inhibitory circuits. J. Neurosci. *26*, 8691–8701.

Kanold, P.O., and Luhmann, H.J. (2010). The subplate and early cortical circuits. Annu. Rev. Neurosci. *33*, 23–48.

Kanold, P.O., and Shatz, C.J. (2006). Subplate neurons regulate maturation of cortical inhibition and outcome of ocular dominance plasticity. Neuron *51*, 627–638.

Kanold, P.O., Kara, P., Reid, R.C., and Shatz, C.J. (2003). Role of subplate neurons in functional maturation of visual cortical columns. Science *301*, 521–525.

Kasper, E.M., Lübke, J., Larkman, A.U., and Blakemore, C. (1994). Pyramidal neurons in layer 5 of the rat visual cortex. III. Differential maturation of axon targeting, dendritic morphology, and electrophysiological properties. J. Comp. Neurol. *339*, 495–518.

Katz, L.C., and Shatz, C.J. (1996). Synaptic activity and the construction of cortical circuits. Science *274*, 1133–1138.

Kätzel, D., and Miesenböck, G. (2014). Experience-dependent rewiring of specific inhibitory connections in adult neocortex. PLoS Biol. *12*, e1001798.

Kätzel, D., Zemelman, B.V., Buetfering, C., Wölfel, M., and Miesenböck, G. (2011). The columnar and laminar organization of inhibitory connections to neocortical excitatory cells. Nat. Neurosci. *14*, 100–107.

Khazipov, R., Sirota, A., Leinekugel, X., Holmes, G.L., Ben-Ari, Y., and Buzsáki, G. (2004). Early motor activity drives spindle bursts in the developing somatosensory cortex. Nature *432*, 758–761.

Koester, S.E., and O'Leary, D.D. (1992). Functional classes of cortical projection neurons develop dendritic distinctions by class-specific sculpting of an early common pattern. J. Neurosci. *12*, 1382–1393.

König, N., Roch, G., and Marty, R. (1975). The onset of synaptogenesis in rat temporal cortex. Anat. Embryol. (Berl.) *148*, 73–87.

Li, H., Fertuzinhos, S., Mohns, E., Hnasko, T.S., Verhage, M., Edwards, R., Sestan, N., and Crair, M.C. (2013). Laminar and columnar development of barrel cortex relies on thalamocortical neurotransmission. Neuron *79*, 970–986.

Luhmann, H.J., Kilb, W., and Hanganu-Opatz, I.L. (2009). Subplate cells: amplifiers of neuronal activity in the developing cerebral cortex. Front. Neuroanat. *3*, 19.

Luhmann, H.J., Kirischuk, S., Sinning, A., and Kilb, W. (2014). Early GABAergic circuitry in the cerebral cortex. Curr. Opin. Neurobiol. *26*, 72–78.

Matsui, A., Tran, M., Yoshida, A.C., Kikuchi, S.S., U, M., Ogawa, M., and Shimogori, T. (2013). BTBD3 controls dendrite orientation toward active axons in mammalian neocortex. Science *342*, 1114–1118.

Michailov, G.V., Sereda, M.W., Brinkmann, B.G., Fischer, T.M., Haug, B., Birchmeier, C., Role, L., Lai, C., Schwab, M.H., and Nave, K.A. (2004). Axonal neuregulin-1 regulates myelin sheath thickness. Science *304*, 700–703.

Miesenböck, G. (2011). Optogenetic control of cells and circuits. Annu. Rev. Cell Dev. Biol. *27*, 731–758.

Minlebaev, M., Ben-Ari, Y., and Khazipov, R. (2007). Network mechanisms of spindle-burst oscillations in the neonatal rat barrel cortex in vivo. J. Neurophysiol. *97*, 692–700.

Minlebaev, M., Ben-Ari, Y., and Khazipov, R. (2009). NMDA receptors pattern early activity in the developing barrel cortex in vivo. Cereb. Cortex *19*, 688–696.

Minlebaev, M., Colonnese, M., Tsintsadze, T., Sirota, A., and Khazipov, R. (2011). Early γ oscillations synchronize developing thalamus and cortex. Science *334*, 226–229.

Miyoshi, G., Butt, S.J., Takebayashi, H., and Fishell, G. (2007). Physiologically distinct temporal cohorts of cortical interneurons arise from telencephalic Olig2-expressing precursors. J. Neurosci. *27*, 7786–7798.

Owens, D.F., Liu, X., and Kriegstein, A.R. (1999). Changing properties of GABA(A) receptor-mediated signaling during early neocortical development. J. Neurophysiol. *82*, 570–583.

Pangratz-Fuehrer, S., and Hestrin, S. (2011). Synaptogenesis of electrical and GABAergic synapses of fast-spiking inhibitory neurons in the neocortex. J. Neurosci. *31*, 10767–10775.

Piñon, M.C., Jethwa, A., Jacobs, E., Campagnoni, A., and Molnár, Z. (2009). Dynamic integration of subplate neurons into the cortical barrel field circuitry during postnatal development in the Golli-tau-eGFP (GTE) mouse. J. Physiol. *587*, 1903–1915.

Porter, J.T., Johnson, C.K., and Agmon, A. (2001). Diverse types of interneurons generate thalamus-evoked feedforward inhibition in the mouse barrel cortex. J. Neurosci. *21*, 2699–2710.

Pouchelon, G., Gambino, F., Bellone, C., Telley, L., Vitali, I., Lüscher, C., Holtmaat, A., and Jabaudon, D. (2014). Modality-specific thalamocortical inputs instruct the identity of postsynaptic L4 neurons. Nature *511*, 471–474.

Raastad, M., Storm, J.F., and Andersen, P. (1992). Putative single quantum and single fibre excitatory postsynaptic currents show similar amplitude range and variability in rat hippocampal slices. Eur. J. Neurosci. *4*, 113–117.

Sadaka, Y., Weinfeld, E., Lev, D.L., and White, E.L. (2003). Changes in mouse barrel synapses consequent to sensory deprivation from birth. J. Comp. Neurol. *457*, 75–86.

Salami, M., Itami, C., Tsumoto, T., and Kimura, F. (2003). Change of conduction velocity by regional myelination yields constant latency irrespective of distance between thalamus and cortex. Proc. Natl. Acad. Sci. USA *100*, 6174–6179.

Spiegel, I., Mardinly, A.R., Gabel, H.W., Bazinet, J.E., Couch, C.H., Tzeng, C.P., Harmin, D.A., and Greenberg, M.E. (2014). Npas4 regulates excitatory-inhibitory balance within neural circuits through cell-type-specific gene programs. Cell *157*, 1216–1229.

Sugiyama, S., Di Nardo, A.A., Aizawa, S., Matsuo, I., Volovitch, M., Prochiantz, A., and Hensch, T.K. (2008). Experience-dependent transfer of Otx2 homeoprotein into the visual cortex activates postnatal plasticity. Cell *134*, 508–520.

Takesian, A.E., Kotak, V.C., and Sanes, D.H. (2010). Presynaptic GABA(B) receptors regulate experience-dependent development of inhibitory short-term plasticity. J. Neurosci. *30*, 2716–2727.

Takesian, A.E., Kotak, V.C., Sharma, N., and Sanes, D.H. (2013). Hearing loss differentially affects thalamic drive to two cortical interneuron subtypes. J. Neurophysiol. *110*, 999–1008.

Tan, Z., Hu, H., Huang, Z.J., and Agmon, A. (2008). Robust but delayed thalamocortical activation of dendritic-targeting inhibitory interneurons. Proc. Natl. Acad. Sci. USA *105*, 2187–2192.

Tang, Y., Stryker, M.P., Alvarez-Buylla, A., and Espinosa, J.S. (2014). Cortical plasticity induced by transplantation of embryonic somatostatin or parvalbumin interneurons. Proc. Natl. Acad. Sci. USA *111*, 18339–18344.

Taniguchi, H., He, M., Wu, P., Kim, S., Paik, R., Sugino, K., Kvitsiani, D., Fu, Y., Lu, J., Lin, Y., et al. (2011). A resource of Cre driver lines for genetic targeting of GABAergic neurons in cerebral cortex. Neuron *71*, 995–1013.

Tolner, E.A., Sheikh, A., Yukin, A.Y., Kaila, K., and Kanold, P.O. (2012). Subplate neurons promote spindle bursts and thalamocortical patterning in the neonatal rat somatosensory cortex. J. Neurosci. *32*, 692–702.

Tuncdemir, S.N., Wamsley, B., Stam, F.J., Osakada, F., Goulding, M., Callaway, E.M., Rudy, B., and Fishell, G. (2016). Early somatostatin interneuron connectivity mediates the maturation of deep layer cortical circuits. Neuron *89*, this issue, 521–535.

Van der Loos, H., and Woolsey, T.A. (1973). Somatosensory cortex: structural alterations following early injury to sense organs. Science *179*, 395–398.

Verhage, M., Maia, A.S., Plomp, J.J., Brussaard, A.B., Heeroma, J.H., Vermeer, H., Toonen, R.F., Hammer, R.E., van den Berg, T.K., Missler, M., et al. (2000). Synaptic assembly of the brain in the absence of neurotransmitter secretion. Science *287*, 864–869.

Vue, T.Y., Lee, M., Tan, Y.E., Werkhoven, Z., Wang, L., and Nakagawa, Y. (2013). Thalamic control of neocortical area formation in mice. J. Neurosci. *33*, 8442–8453.

Wang, Y., Toledo-Rodriguez, M., Gupta, A., Wu, C., Silberberg, G., Luo, J., and Markram, H. (2004). Anatomical, physiological and molecular properties of Martinotti cells in the somatosensory cortex of the juvenile rat. J. Physiol. *561*, 65–90.

Washbourne, P., Thompson, P.M., Carta, M., Costa, E.T., Mathews, J.R., Lopez-Benditó, G., Molnár, Z., Becher, M.W., Valenzuela, C.F., Partridge, L.D., and Wilson, M.C. (2002). Genetic ablation of the t-SNARE SNAP-25 distinguishes mechanisms of neuroexocytosis. Nat. Neurosci. *5*, 19–26.

West, D.C., Mercer, A., Kirchhecker, S., Morris, O.T., and Thomson, A.M. (2006). Layer 6 cortico-thalamic pyramidal cells preferentially innervate interneurons and generate facilitating EPSPs. Cereb. Cortex *16*, 200–211.

Xu, X., and Callaway, E.M. (2009). Laminar specificity of functional input to distinct types of inhibitory cortical neurons. J. Neurosci. *29*, 70–85.

Xu, H., Jeong, H.Y., Tremblay, R., and Rudy, B. (2013). Neocortical somatostatin-expressing GABAergic interneurons disinhibit the thalamorecipient layer 4. Neuron *77*, 155–167.

Xue, M., Atallah, B.V., and Scanziani, M. (2014). Equalizing excitation-inhibition ratios across visual cortical neurons. Nature *511*, 596–600.

Yang, J.W., Hanganu-Opatz, I.L., Sun, J.J., and Luhmann, H.J. (2009). Three patterns of oscillatory activity differentially synchronize developing neocortical networks in vivo. J. Neurosci. *29*, 9011–9025.

Yang, J.W., An, S., Sun, J.J., Reyes-Puerta, V., Kindler, J., Berger, T., Kilb, W., and Luhmann, H.J. (2013a). Thalamic network oscillations synchronize ontogenetic columns in the newborn rat barrel cortex. Cereb. Cortex *23*, 1299–1316.

Yang, W., Carrasquillo, Y., Hooks, B.M., Nerbonne, J.M., and Burkhalter, A. (2013b). Distinct balance of excitation and inhibition in an interareal feedforward and feedback circuit of mouse visual cortex. J. Neurosci. *33*, 17373–17384.

Zemelman, B.V., Nesnas, N., Lee, G.A., and Miesenbock, G. (2003). Photochemical gating of heterologous ion channels: remote control over genetically designated populations of neurons. Proc. Natl. Acad. Sci. USA *100*, 1352–1357.

Zhao, C., Kao, J.P., and Kanold, P.O. (2009). Functional excitatory microcircuits in neonatal cortex connect thalamus and layer 4. J. Neurosci. *29*, 15479–15488.

Neuron
Supplemental Information

A Transient Translaminar GABAergic Interneuron Circuit Connects Thalamocortical Recipient Layers in Neonatal Somatosensory Cortex

Andre Marques-Smith, Daniel Lyngholm, Anna-Kristin Kaufman,
Jacqueline A. Stacey, Anna Hoerder-Suabedissen, Esther B.E. Becker, Michael C.
Wilson, Zoltán Molnár, and Simon J.B. Butt

SUPPLEMENTAL FIGURES

Figure S1 [related to Figure 3 and Figure 4]. Laminar organisation of normalised glutamatergic input onto Lpar1-INs and GABAergic input onto L4 SSNs

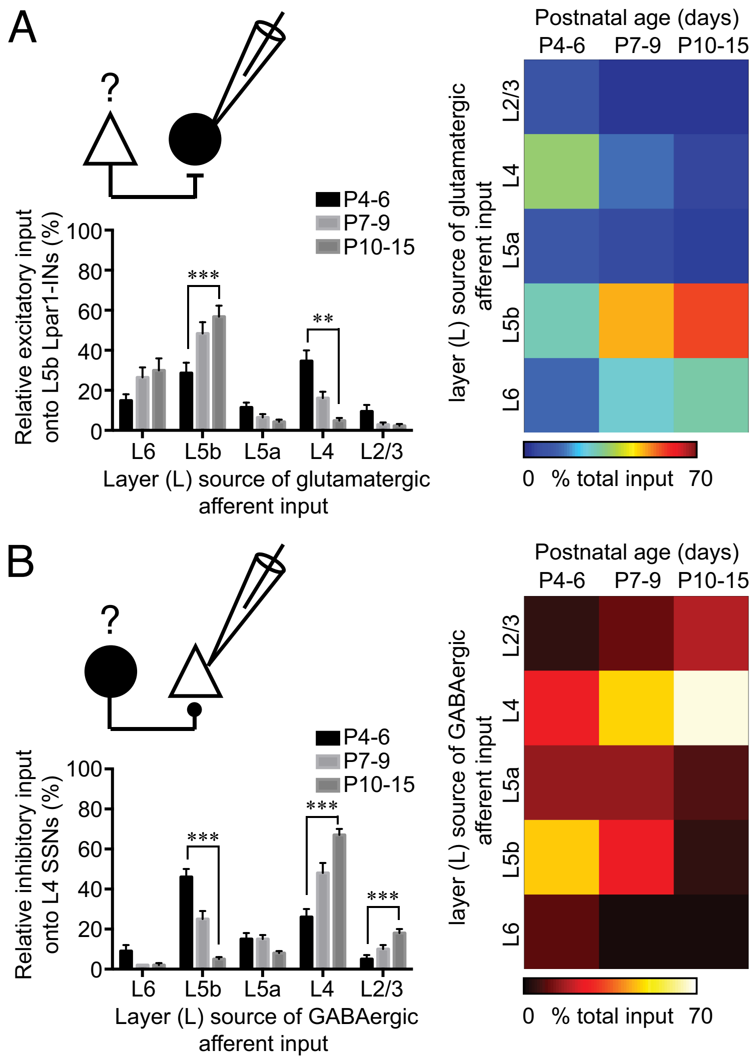

(A) *Left*, percentage afferent excitatory input from each layer onto Lpar1-INs (schematic black circle) as revealed by LSPS of caged glutamate. Local L5b synaptic input increased significantly between P4-6 and P10-15 (Kruskal-Wallis test, H(3, 29) = 9.86, p = 0.007; Dunn's multiple comparisons test (Dmc), p = 0.002). L4 input decreased significantly (Kruskal-Wallis test, H(3, 29) = 13.50, p = 0.001; Dmc, p = 0.0002). Error bars are ±SEM. *Right*, summary plot showing the developmental remodelling of afferent excitatory laminar input sources onto Lpar1-INs through development.
(B) *Left*, percentage afferent GABAergic input per layer revealed by LSPS of caged glutamate and holding the postsynaptic L4 SSN at E_{Glut}. Input originating from L5b decreased significantly between P4-6 and P10-15 (Kruskal-Wallis test, H(3, 51) = 30.9, p = 0.0001; Dunn, p = 0.001), as did L5a input (Kruskal-Wallis test, H(3, 51) = 8.43, p = 0.015; Dunn, p = 0.054). L4 input significantly increased over the same period (Kruskal-Wallis test, F(3, 51) = 31.4, p = 0.0001; Dunn, p = 0.0001), as did L2/3 input (Kruskal-Wallis test, H(3, 51) = 20.3, p = 0.0001; Dunn, p = 0.001). Error bars are +/-SEM. *Right*, summary plot of the developmental remodelling of afferent GABAergic input onto Lpar1-EGFP INs as arranged by laminar sources.

Figure S2 [related to Figure 5]. Infraorbital nerve (ION) transection reduces total glutamatergic input onto L5b Lpar-INs.

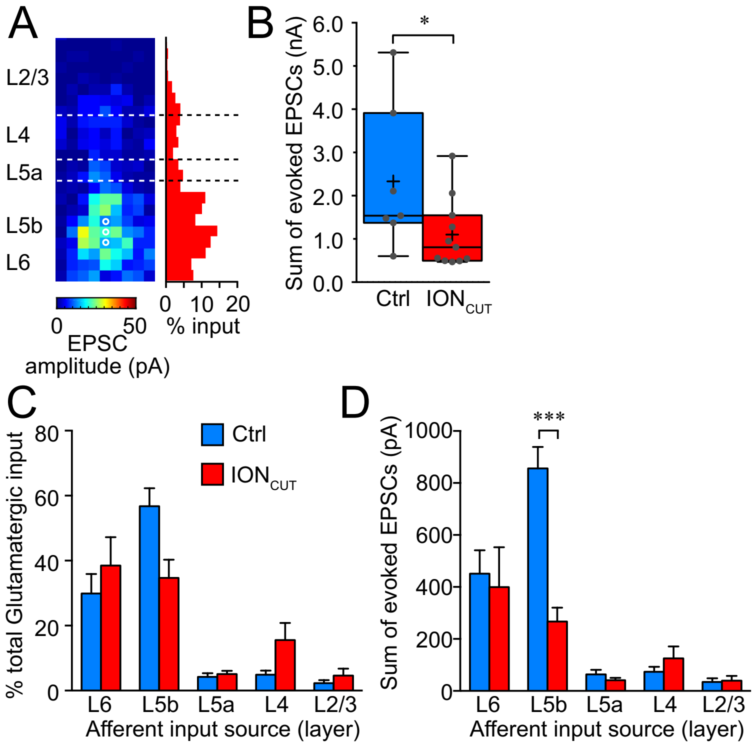

(A) Average map (left) of evoked glutamatergic input onto Lpar1-INs (n=11) in ION transected (ION_{cut}) animals at P10-15. *Right panel*, normalised profile of glutamatergic input onto ION_{cut} Lpar1-INs across the depth of the cortical column.
(B) Total glutamatergic input onto Lpar1-INs at P10-15 is significantly reduced (*p=0.020, Mann-Whitney test, U(82,108)=16) in IONcut animals compared to controls.
(C) Normalised glutamatergic input onto Lpar1-INs showed no layer difference in distribution between IONcut and control at P10-15.
(D) Total glutamatergic input on Lpar1-INs in IONcut animals broken down according to layer. The decrease in total input from L5b (***p<0.0001, Mann-Whitney test, U(69,141)=3.00) was not accompanied by a compensatory increase in other cortical layers.

Figure S3 [related to Figure 6]. Experimental strategy employed to examine the impact of conditionally silencing SST+ cells on the transient L5b-L4 GABAergic connection.

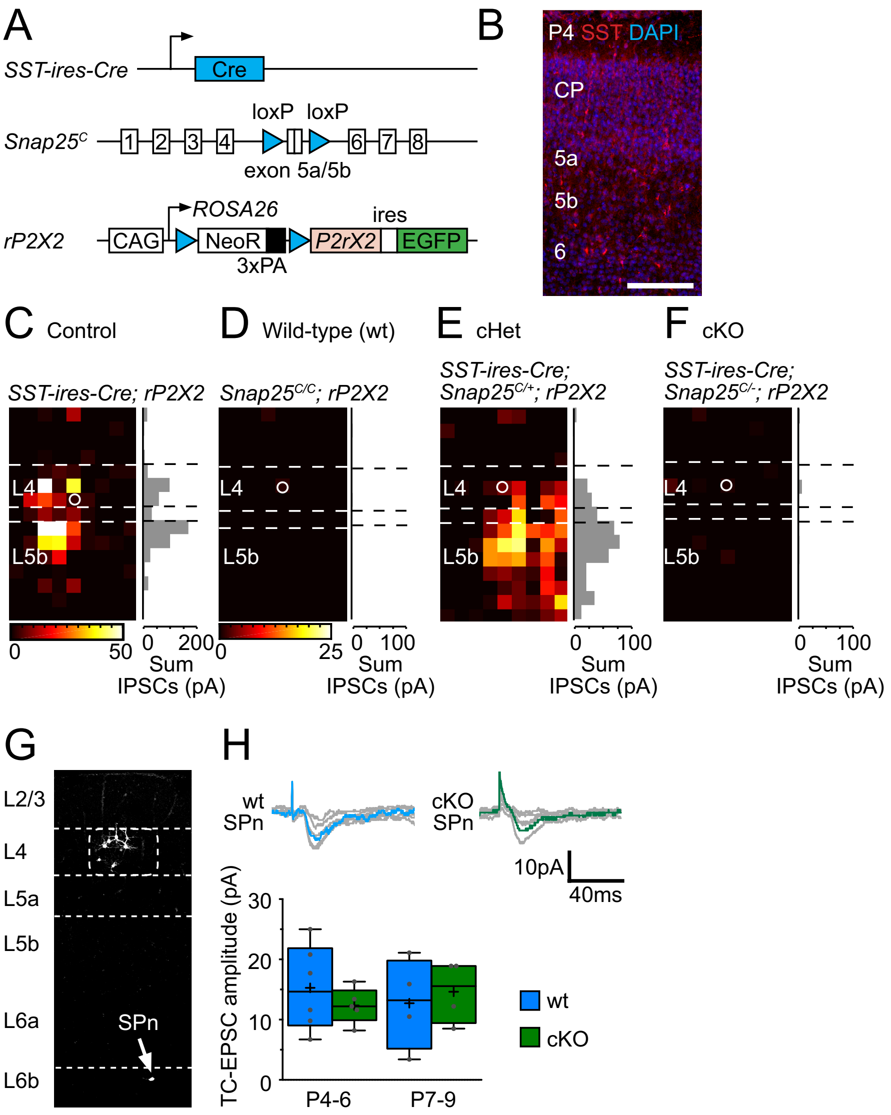

(A) The genetic strategy for examining Snap25 conditional loss-of-function while simultaneously enabling LSPS stimulation of SST+ cells. Males with the SST-ires-Cre driver and a single copy of the floxed Snap25 (*Snap25*^{C}) allele were crossed to females that were homozygote for the floxed Snap25 allele and a floxed-stop rat P2X2 receptor (rP2X2) with bicistronic ires-EGFP for genetic fate mapping. Unfortunately EGFP expression was not detected in SST+ cells either by visual inspection in acute *in vitro* slices or following immunohistochemistry.
(B) The presence of SST+ cells in P4 somatosensory cortex in conditional loss-of-function Snap25 (cKO) animals.
(C-F) Use of the P2X2 optogenetic strategy enables us to determine the source of SST+ cell input onto SSNs in the developing neocortex and confirm an absence of SST+ IN signalling in Snap25 cKO pups.
(C) LSPS map data from a P5 SSN in a control animal conditionally expressing rP2X2 in cells expressing Cre under the SST-ires-Cre driver line. UV laser uncaging of ATP evoked action potentials in cells expressing the rP2X2 receptor enabling LSPS mapping of input onto the SSN (left panel). Right panel, distribution of input onto the SSN arising from SST+ cells. Dashed white lines, layer boundaries; white circle, cell location.
(D) No GABAergic input was mapped onto L4 SSNs following ATP uncaging in animals that did not express Cre. The example shown is a recording from a L4 SSN (white circle) in an acute *in vitro* slice preparation of a P5 Snap25$^{C/C}$;rP2X2 pup.

(*Cont.*)

(E) LSPS map of ATP uncaging-evoked responses onto a L4 SSN in a P6 conditional Snap25 heterozygote (cHet) pup. Scale bar is the same as shown in panel (D).
(F) Failure to observe ATP uncaging-evoked GABAergic inputs onto a L4 SSN in a conditional Snap25 knockout (cKO) P5 animal.
(G) Recovered morphologies of L4 SSNs and a single nearby subplate neuron (SPn) in a P7 wild-type acute *in vitro* thalamocortical slice. Recordings of L4 SSNs were only performed in animals in which thalamocortical connectivity into the neocortex was confirmed by preservation of input onto SPns (see panel H).
(H) Electrical stimulation of the VPM evoked TC-EPSCs in subplate (SPn) neurons in both wild-type (wt; blue average trace) and Snap25 conditional knockout (cKO) animals (both P7). *Bottom graph*, amplitude of TC-EPSCs recorded under voltage clamp (hp: -70mV) in SPn.

Figure S4 [related to Figure 7]. Over-expression of Nrg-1type1 protein in early postnatal development does not result in a change of spontaneous synaptic activity recorded from L4 neurons.

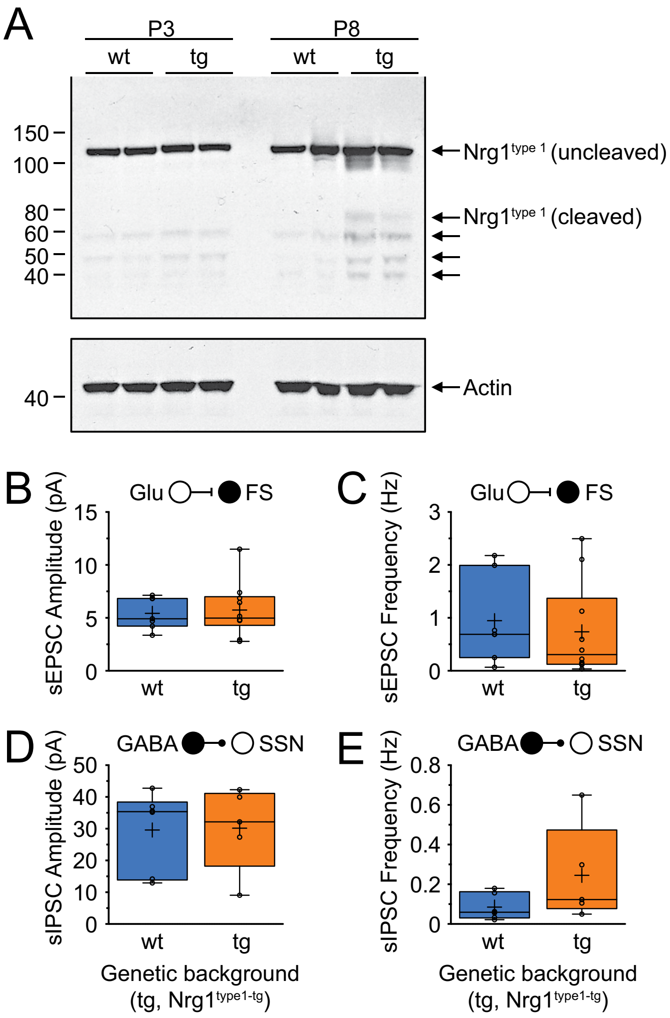

(A) Western blot for Ig-Neuregulin-1 (type 1) using cortical tissue from P3 and P8 animals in wild-type (wt) and over-expression Nrg-1type1-tg (tg) littermates. β Actin was used as loading control.
(B) Amplitude of spontaneous excitatory postsynaptic currents (sEPSCs)(hp:-70mV) recorded in layer 4 fast spiking (FS) interneurons arising from glutamatergic (Glu) afferent input; wt, n=7 cells from 6 animals (P4-P7); tg, n=10 cells from 9 animals (P4-P7).
(C) Frequency of sEPSCs (hp:-70mV) recorded in the same FS INs as panel (A)
(D,E) Corresponding data for spontaneous inhibitory postsynaptic currents recorded in L4 spiny stellate neurons (SSNs) arising from afferent input from GABAergic INs; wt, n=6 cells from 4 animals (P4-P7); tg, n=6 cells from 4 animals (P4-P7).

SUPPLEMENTAL TABLES

Table S1 [related to Figure 1]. Maturation of the intrinsic electrophysiological properties of S1BF Lpar1-INs.

	P4-P6 (n = 12)	P7-P9 (n = 14)	P10-P15 (n = 29)
**RMP (mV)**	-58.0 ±2.1	-56.3 ±1.0	-56.6 ±0.8
*****R_{in} (MΩ)**	1349 ±109	529 ±56	372 ±51
*****Rheobase (pA)**	8± 1.0	17 ±2	30± 4
*****Tau (ms)**	78.5 ±5.4	36.4 ±3.4	31.5 ±3.0
***AP threshold (mV)**	-39.5 ±1.0	-43.0 ±0.9	-42.7 ±3.1
****AP height (mV)**	58.5 ±2.8	68.7 ±2.9	69.6 ±1.7
*****AP HW (ms)**	3.0±0.2	1.8±0.1	1.3±0.1
***Adaptation (%)**	19± 5	22± 3	30± 2
***sAHP time (ms)**	37.1± 3.7	24.2± 2.7	25.9± 3.1
**sAHP amp. (mV)**	14.1± 0.5	13.7± 0.8	13.5± 1.1
**fAHP time (ms)**	-	-	5.9± 0.4
**fAHP amp. (mV)**	-	-	11.1± 1.2
**Voltage sag (mV)**	1.8± 1.6	5.5± 0.7	6.4± 0.50
*****Max. Freq. (Hz)**	38± 3	53± 3	70± 4

Table S1 abbreviations: RMP, resting membrane potential; R_{in}, input resistance; AP, action potential; AHP, after-hyperpolarisation. Values reported in each cell are average± SEM. Asterisks indicate one-way ANOVA or Kruskal-Wallis test *p* values: * $p< 0.05$; ** $p< 0.01$; *** $p< 0.001$.

Table S2 [related to Figure 5]. Intrinsic excitability of L4 fast-spiking and L5b Lpar1-INs in control and ION_{CUT} groups at P10-P15.

	L4 FS INs P10-P15		L5b Lpar1-INs P10-P15	
	**Control n = 15**	**ION_{CUT} n = 8**	**Control n = 29**	**ION_{CUT} n = 12**
**RMP (mV)**	-60 ± 1	-62 ± 2	-57 ±1	-59 ± 1
**R_{in} (MΩ)**	194 ± 16	281 ± 26*	372 ±51	455 ± 73
**Rheobase (pA)**	127 ± 20	81 ± 25	30± 4	21 ± 4
**AP threshold (mV)**	-31 ± 1	-32 ± 1	-43 ±3	-43 ± 1
**Max. Freq. (Hz)**	122 ± 8	101 ± 13	70± 4	79 ± 4
**Tau (ms)**	26 ± 4	17 ± 1	32 ±3	30 ± 2

Table S2 abbreviations as in Table S1. Values reported in each cell are mean ±SEM. Asterisks indicate Student's t test or Mann-Whitney test significance *p* values: ** $p< 0.01$. Statistically significant differences were only found for Input Resistance between Control and ION_{CUT} L4 FS INs (Student's t test, t (21) = 2.82, $p = 0.010$).
